# Early Cardiac Rehabilitation for Critically Ill Patients With Acute Decompensated Heart Failure: A Randomized Clinical Trial

**DOI:** 10.1001/jamanetworkopen.2025.24141

**Published:** 2025-07-30

**Authors:** Linjing Wu, Jiahua Li, Yamin Zheng, Mengmeng Xue, Wei Yan, Yongbin Sun, Meiling Zhang, Qiaoyan Li, Jiahong Zhang, Ying Jia, Yuli Wang, Yuan Chen, Guangyu Sun, Binbin Liu, Cuilian Dai

**Affiliations:** 1 Xiamen Cardiovascular Hospital of Xiamen University, School of Medicine, Fujian Branch of National Clinical Research Center for Cardiovascular Diseases, Xiamen, China

## Abstract

**Importance:**

The optimal timing and approach for initiating cardiac rehabilitation (CR) in critically ill patients during the acute phase of acute decompensated heart failure (ADHF) remains uncertain.

**Objective:**

To evaluate the effects of CR on physical function and rehospitalization for critically ill patients with ADHF admitted to the cardiac intensive care unit (CICU).

**Design, Setting, and Participants:**

In this single-center, single-blind randomized clinical trial conducted in China, critically ill patients with severe ADHF admitted to the CICU were recruited between March 26, 2021, and September 1, 2022. All patients were followed up for 6 months, and investigators were blinded to the group assignment.

**Interventions:**

After short-term therapy, participants were randomized 1:1 to an early progressive and personalized CR program for patients with ADHF (AHF-CR program) that was administered exclusively during the patients’ CICU stay or to usual care.

**Main Outcomes and Measures:**

The primary outcomes were Short Physical Performance Battery (SPPB) score at hospital discharge and 6-month all-cause rehospitalization rates. These outcomes were analyzed using an intention-to-treat approach including all patients after randomization. The Perme Intensive Care Unit Mobility (PERME) score was incorporated as an exploratory outcome during analysis to assess mobility status in critically ill patients.

**Results:**

This study included 120 patients (mean [SD] age, 68.6 [12.3] years; 80 [66.7%] male). At randomization, pulmonary crackles were observed in 49 patients in the control group (81.7%) and 43 patients in the intervention group (71.7%). Additionally, 62 patients (51.7%) had an arterial partial pressure of oxygen to fraction of inspired oxygen ratio below 300 mm Hg. A total of 40 patients (33.3%) received intravenous vasoactive medications, and 87 (72.5%) received intravenous loop diuretics. The median difference in SPPB scores between groups was 1.0 (95% CI, 0-2.0; *P* = .16), which was not significant. Six-month rehospitalization rates were comparable between the control and intervention groups (16 [26.6%] vs 17 [28.3%]; hazard ratio, 1.00 [95% CI, 0.51-1.99]; *P* = .99). Exploratory analysis revealed that the intervention group had higher PERME scores, with a median between-group difference of 2.76 (95% CI, 0.77-4.74; adjusted *P* = .04).

**Conclusions and Relevance:**

In this randomized clinical trial of critically ill patients with ADHF, the AHF-CR program did not significantly improve SPPB scores or rehospitalization rates. However, it may offer potential physical benefits, including enhanced mobility.

**Trial Registration:**

Chinese Clinical Trial Registry Identifier: ChiCTR2100050151

## Key Points

**Question:**

Are there benefits of early cardiac rehabilitation for physical function and rehospitalization for critically ill patients with acute decompensated heart failure (ADHF) admitted to the cardiac intensive care unit?

**Findings:**

In this randomized clinical trial involving 120 critically ill patients with ADHF, there were no significant differences in Short Physical Performance Battery (SPPB) scores or rehospitalization rates between the group receiving cardiac rehabilitation and the group receiving usual care.

**Meaning:**

An early, personalized cardiac rehabilitation program did not alter SPPB scores or rehospitalization rates for critically ill patients with ADHF compared with usual care.

## Teaser

This randomized clinical trial examines the effects of early cardiac rehabilitation on physical function and rehospitalization for critically ill patients with acute decompensated heart failure in the cardiac intensive care unit.

## Introduction

Heart failure (HF) is a major global health burden, with acute decompensated heart failure (ADHF) characterized by sudden worsening of symptoms, fluid overload, and organ dysfunction due to hypoperfusion.^1^ Despite advances in treatment, ADHF continues to be a major cause of morbidity and mortality.^2^ Exercise-based cardiac rehabilitation (CR) has been shown to enhance cardiorespiratory function, exercise tolerance, and quality of life in patients with chronic HF.^3,4^ Early rehabilitation typically involves physical exercise performed within 2 to 5 days for critically ill patients. However, patients in the acute phase of ADHF, particularly those with hemodynamic instability or impaired heart function classified as New York Heart Association (NYHA) functional class IV, have contraindications for exercise therapy or are considered at increased risk.^5,6^ Identifying the optimal timing and strategy for initiating a progressive, individualized CR program in patients with severe ADHF, particularly those in the critical illness stage during their stay in the cardiac intensive care unit (CICU), is crucial for improving clinical outcomes.

Impaired physical function and frailty are prevalent among adults with HF, particularly in older patients,^7^ and are associated with increased risks of mortality and hospitalization.^8,9^ Previous randomized clinical trials (RCTs) on early CR for patients with ADHF have demonstrated benefits for physical function. However, these studies primarily focused on hospitalized patients and excluded those in the acute phase, particularly individuals still receiving intensive care in the CICU.^10,11,12^ Patients in ICUs lose approximately 1.75% of their rectus femoris thickness or 2.10% of their rectus femoris cross-sectional area daily,^13^ indicating that ICU-acquired weakness remains a common complication of critical illness significantly impacting morbidity and mortality.^14^ Early physical and occupational therapy for critically ill ICU patients receiving mechanical ventilation has shown some functional improvements.^15,16^ Based on evidence from ICU patients without ADHF, early mobilization is recommended for CICU patients to prevent complications.^17^ However, some studies suggest that increased early active mobilization for ICU patients receiving mechanical ventilation and those with acute respiratory failure does not lead to significantly greater improvements in hospital length of stay or post-ICU outcomes.^18,19^ Therefore, the benefits of a CR program for patients with severe ADHF in the CICU remain largely unknown.

In this study, we conducted a single-center, single-blind RCT involving patients with severe ADHF (classified as NYHA functional class III or IV) who were hospitalized in the CICU. An early, progressive, and personalized CR program for patients with ADHF, termed AHF-CR, was implemented exclusively during the CICU stay. Our investigation focused on physical function, including mobility status, health-related quality of life, and self-maintenance abilities, and end point events, such as rehospitalization and mortality rates, to evaluate the safety and efficacy of CR for patients with severe ADHF during the acute phase in the CICU.

## Methods

### Study Protocol

The trial design and protocol have been previously published^20^ and are given in Supplement 1. This RCT was conducted at Xiamen Cardiovascular Hospital of Xiamen University in Xiamen, China (ChiCTR2100050151). The investigation adhered to the principles outlined in the Declaration of Helsinki.^21^ The protocol was approved by the Ethics Committee of Xiamen Cardiovascular Hospital of Xiamen University, and written informed consent was obtained from all participants. The study design and result reporting followed the Consolidated Standards of Reporting Trials (CONSORT) reporting guideline for RCTs.

Eligible participants were adults aged 18 years or older who were admitted to the CICU with severe ADHF classified as NYHA class III or IV, including patients with cardiogenic shock and those with regurgitation and stenosis of the mitral, tricuspid, and aortic valves (excluding severe aortic valve stenosis). Participants presented with exacerbated symptoms, including dyspnea, systemic venous congestion, or tissue hypoperfusion, and required mechanical or noninvasive ventilation. Patients were excluded if they had acute coronary syndrome within the past 2 days; had onset of chest pain within 8 hours; had fatal arrhythmia, severe aortic valve stenosis, aortic dissection, acute myocarditis, acute pericarditis, acute infective endocarditis, recent embolism, atrial or ventricular thrombosis, or thrombophlebitis; were undergoing long-term hemodialysis; had an inability to walk without assistance; or exhibited cognitive impairment prior to the acute CICU illness. All patients received standard therapy prior to randomization. This therapy followed the European Society of Cardiology guidelines and aimed to stabilize hemodynamic status, correct hypoxemia, and maintain organ perfusion and function; identify and manage the underlying causes and triggers of acute HF to prevent recurrence; alleviate HF symptoms; prevent acute decompensation; and ultimately improve patients’ quality of life and long-term prognosis.^22,23^

Randomization and physical therapy occurred only after patients met the following criteria: no onset or recurrence of chest pain in the past 8 hours, no new symptoms of decompensated HF, no new arrhythmias or dynamic changes on electrocardiography within the past 8 hours, no further elevation in troponin levels, no increases in vasoactive medication doses, a resting heart rate less than 110 beats per minute, resting systolic blood pressure of 90 to 150 mm Hg and diastolic blood pressure of 60 to 100 mm Hg, and blood oxygen saturation (Sao_2_) of 92% or greater. No stratification was applied during the randomization process. Participants were randomly assigned to a control group receiving usual care or an intervention group receiving AHF-CR. Blinded physiotherapists conducted daily physical fitness assessments based on muscle strength, consciousness, and NYHA class, assigning fitness levels from 1 to 7 (eTable 1 in Supplement 2). Usual care in the CICU included routine management during the decompensated phase, such as bed rest and standard pneumatic therapy. In the postimprovement phase, patients progressed to active movements, including passive and active limb exercises as well as diaphragmatic and pursed-lip breathing exercises, and gradually transitioned to out-of-bed activities. The AHF-CR program included resistance training, treadmill training, and standing and sitting exercises tailored to each participant’s fitness level (eTable 1 in Supplement 2). To advance to a higher level, patients had to meet the next level’s fitness criteria and complete the training goals of their current level. Physical therapy was discontinued under any of the following conditions: newly developed arrhythmia, an increase in heart rate of 20 beats per minute or more, or dynamic electrocardiographic changes; a systolic blood pressure fluctuation exceeding ±40 mm Hg; Sao_2_ below 92%; a sustained decrease in stroke volume lasting more than 1 minute; or the emergence of exercise-intolerance symptoms, such as chest tightness, palpitations, or shortness of breath. Physical therapy was conducted by trained physiotherapists who were aware of group assignments. Physical therapy ceased at CICU discharge, with no specific exercise recommendations provided after discharge. During 6-month follow-up, patients received monthly telephone calls after hospital discharge to monitor symptoms and clinical events.

### Randomization and Blinding

Patients were randomly assigned using a sequence generated by an independent statistician. Allocation concealment was achieved with sequentially numbered, opaque envelopes. A researcher allocated participants in a 1:1 ratio. Physical therapy was delivered by trained physiotherapists aware of group assignments, while outcome and physical fitness assessments were conducted by researchers blinded to group allocation.

### Outcomes

The primary outcomes were Short Physical Performance Battery (SPPB) score^24,25^ at hospital discharge and 6-month all-cause rehospitalization. The SPPB evaluates physical function using the standing balance test, a 4-m gait speed test, and a strength test (time to rise from a chair 5 times), with scores ranging from 0 to 12; lower scores indicate greater physical dysfunction.^24,25^ Six-month all-cause rehospitalization included deaths occurring during rehospitalization but excluded deaths that occurred at home within 6 months after discharge. Secondary outcomes included 6-month all-cause mortality, activities of daily living (ADLs) to measure self-maintenance abilities at CICU discharge,^26^ and the Physical Component Summary (PCS) score from the 36-Item Short Form Survey (SF-36) at 6 months after hospital discharge.^27^ The ADL scale measures self-maintenance abilities, including basic daily tasks, mobility, and instrumental activities of daily living, with scores ranging from 0 to 100; higher scores indicate greater self-maintenance capacity.^26^ The SF-36 is a widely used instrument for assessing health-related quality of life. The PCS score, derived from the SF-36, evaluates physical health, with higher scores indicating better physical functioning.^27^ The Perme ICU Mobility (PERME) score^28,29^ was incorporated into the outcomes during analysis. The PERME score, ranging from 0 to 32, assesses impairment and activity limitations in critically ill patients. It evaluates mental status, mobility barriers, functional strength, bed mobility, transfers, gait, and endurance, with higher scores indicating fewer limitations. Collectively, the primary and secondary outcomes, including SPPB, ADL, PCS, and PERME scores, primarily focused on evaluating the physical performance of the patients. Cardiopulmonary function was assessed at CICU discharge as a supporting outcome. The in-hospital mortality rate was not calculated due to the limited number of cases.

### Statistical Analysis

The sample size for each group was determined based on findings from a previous study,^12^ in which baseline mean (SD) SPPB scores were 6.0 (2.8) in the intervention group and 6.1 (2.6) in the control group. At 3 months, mean (SD) scores were 8.3 (0.2) and 6.9 (0.2), respectively.^12^ Based on these data, the pooled SD (σ) was estimated to be approximately 1.9, and the expected mean difference (μ_1_ − μ_2_) was 1.5. The sample size was calculated using the following standard z test formula: n = 2σ^2^[Z_1−α/2_ + Z_1−β_]^2^ / (μ_1_ − μ_2_)^2^. To minimize both false-positive and false-negative errors, we set the type I error rate (α) at .01 and the type II error rate (β) at .10. Additionally, a 20% dropout rate and 7% missing data were anticipated, resulting in a final sample size of 60 participants per group. No power analysis was conducted for the coprimary outcome of all-cause rehospitalization.

All outcomes were analyzed using the intention-to-treat population, which included all randomized patients. No covariate adjustments were prespecified in our study. Missing data were handled with multiple imputation by chained equations incorporating variables such as group, age, sex, body mass index, smoking and alcohol history, baseline PERME scores, and intervention assessments. Combined probabilities were pooled using the Rubin rules. SPPB scores were analyzed using the Mann-Whitney *U* test with the Hodges-Lehmann method for median difference and *P* values. ADL, PCS, and cardiopulmonary function outcomes as well as PERME scores in subgroups were compared using the Wilcoxon rank-sum test based on changes between baseline and final scores. The PERME score difference was analyzed with analysis of covariance adjusting for baseline levels, and assumptions were verified. Cox proportional hazards regression analysis estimated unadjusted hazard ratios (HRs) and *P* values for time-to-event outcomes, and Kaplan-Meier curves are presented. The proportional hazards assumption was assessed using log-minus-log plots. Adjusted *P* values for primary and secondary outcomes were calculated using the Holm-Bonferroni method applying the correction across all tests. The 95% CIs were not adjusted for multiplicity. PERME scores and cardiopulmonary function outcomes were considered exploratory, as they were not prespecified trial outcomes. Data analyses were performed using SPSS, version 29.0 (SPSS Inc) or R, version 4.4.1 (R Project for Statistical Computing). Two-sided *P* < .05 was considered significant.

## Results

### Recruitment, Physical Therapy, and Follow-Up

**Figure 1.**
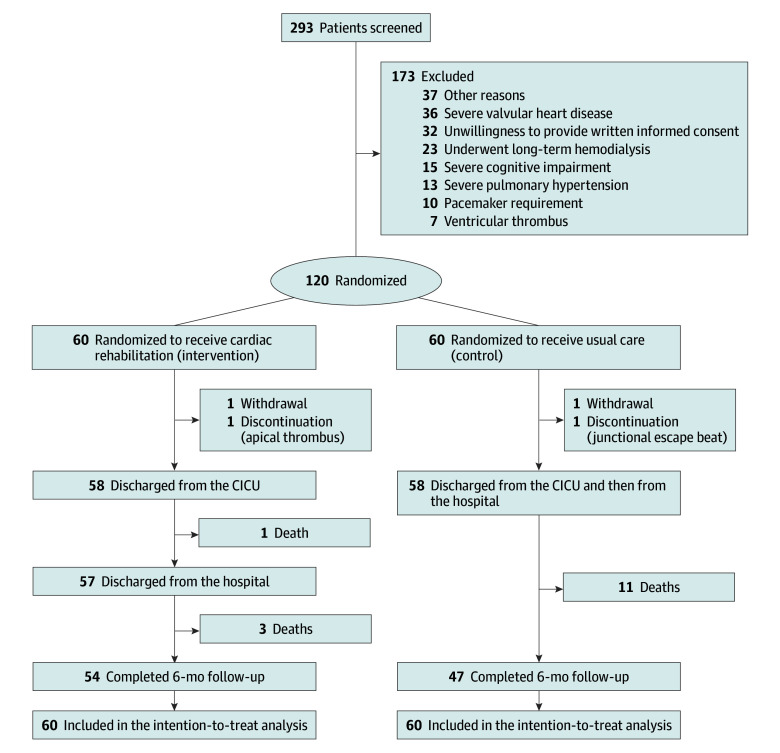
Flow of Patients Through the Study Before randomization, all patients with severe acute decompensated heart failure in the cardiac intensive care unit (CICU) received standard therapy to meet the following criteria: no onset or recurrence of chest pain in the past 8 hours; no new symptoms of decompensated heart failure; no new arrhythmias or dynamic changes on electrocardiography within the past 8 hours; no further elevation in troponin levels; no increases in vasoactive mediation doses; a resting heart rate of less than 110 beats per minute; resting systolic blood pressure of 90 to 150 mm Hg and diastolic blood pressure of 60 to 100 mm Hg; and blood oxygen saturation of 92% or greater. In the control group, usual care consisted of the standard level of mobilization typically provided in each CICU.

A total of 293 patients with severe ADHF were screened at the CICU from March 26, 2021, to September 1, 2022. Of these, 173 patients were excluded prior to randomization (Figure 1). Ultimately, 120 patients met the inclusion criteria and were randomized to the control or intervention group (n = 60 each). Patients had a mean (SD) age of 68.6 (12.3) years; there were 40 females (33.3%) and 80 males (66.7%) (Table 1). During the study, 2 patients withdrew consent but allowed the use of their data, while 2 discontinued due to severe complications: one experienced junctional escape beats, and the other developed an apical thrombus. However, all participants were followed up for 6 months and were included in the intention-to-treat analysis (Figure 1). Consequently, 4 patients had missing data for SPPB, PERME, or SF-36 scores, accounting for 3.3% of the total dataset.

**Table 1.** Characteristics of Patients at Baseline

Characteristic	Patients^a^		
	Total (N = 120)	Control (n = 60)	Intervention (n = 60)
Age, mean (SD), y	68.6 (12.3)	70.2 (13.4)	66.9 (11.0)
Sex			
Female	40 (33.3)	22 (36.7)	18 (30.0)
Male	80 (66.7)	38 (63.3)	42 (70.0)
Height, mean (SD), cm	163.1 (8.1)	162.3 (8.2)	163.9 (8.1)
Body weight, mean (SD), kg	64.0 (12.9)	61.5 (12.2)	66.5 (13.3)
BMI, median (IQR)	24.0 (20.9-26.4)	22.4 (20.2-24.9)	24.8 (24.2-26.7)
Pulmonary crackles	92 (76.7)	49 (81.7)	43 (71.7)
Heart rate, mean (SD), beats/min	83.3 (13.8)	83.4 (14.4)	83.3 (13.2)
Systolic blood pressure, mean (SD), mm Hg	120.6 (18.5)	120.2 (18.6)	121.0 (18.5)
Diastolic blood pressure, mean (SD), mm Hg	70.0 (11.7)	69.3 (12.7)	70.6 (10.6)
Ventilation			
Mechanical	3 (2.5)	1 (1.7)	2 (3.3)
Noninvasive	117 (97.5)	59 (98.3)	58 (96.7)
Smoking history			
Never	51 (42.5)	27 (45.0)	24 (40.0)
Former	35 (29.2)	15 (25.0)	20 (33.3)
Current	34 (28.3)	18 (30.0)	16 (26.7)
Alcohol history			
Never	85 (70.8)	47 (78.3)	38 (63.3)
Former	9 (7.5)	2 (3.3)	7 (11.7)
Current	26 (21.7)	11 (18.3)	15 (25.0)
Etiology			
Ischemic cardiomyopathy	61 (50.8)	34 (56.7)	27 (45.0)
Valvular diseases^b^	10 (8.3)	5 (8.3)	5 (8.3)
Hypertension	6 (5.0)	2 (3.3)	4 (6.7)
Atrial fibrillation	8 (6.7)	6 (10.0)	2 (3.3)
Dilated cardiomyopathy	15 (12.5)	4 (6.7)	11 (18.3)
Other	20 (16.7)	9 (15.0)	11 (18.3)
NYHA functional class			
II^c^	2 (1.7)	2 (3.3)	0
III	64 (53.3)	32 (53.3)	32 (53.3)
IV	54 (45.0)	26 (43.3)	28 (46.7)
LVEF ≥40%	46 (38.3)	22 (36.7)	24 (40.0)
Echocardiographic findings			
LVEF, mean (SD), %	37.0 (12.9)	36.9 (13.3)	37.1 (12.5)
Left atrial diameter, median (IQR), mm	46 (40-49)	46 (43-51)	53 (48-59)
Interventricular septal thickness at end diastole, mean (SD), mm	9.8 (2.0)	9.7 (2.0)	9.8 (2.1)
Left ventricular end diastolic diameter, mean (SD), mm	60.4 (10.5)	59.8 (11.0)	60.9 (10.1)
Left ventricular end systolic diameter, mean (SD), mm	48.2 (13.1)	48.3 (13.4)	48.1 (12.9)
Left ventricular posterior wall thickness at end diastole, median (IQR), mm	9.4 (8.1-10.0)	9 (7.4-9.9)	9.1 (9-9.7)
Laboratory findings			
Pao_2_:FIo_2_ <300 mm Hg	62 (51.7)	30 (50.0)	32 (53.3)
N-terminal pro–B-type natriuretic peptide, median (IQR), ng/L	5272 (2394-15 876)	5193 (2675-10 537)	3550 (1393-7729)
High-sensitivity cardiac troponin T, median (IQR), ng/L	52.3 (30.1-135.2)	52.1 (30.5-221.0)	54.5 (30.0-122.7)
High-sensitivity C-reactive protein, median (IQR), mg/dL	1.10 (0.32-3.06)	1.88 (0.59-2.75)	0.78 (0.19-1.52)
Triglycerides, median (IQR), mg/dL	88.5 (70.8-115.0)	79.6 (62.0-97.4)	88.5 (70.8-115.0)
Cholesterol, mean (SD), mg/dL	146.7 (46.3)	146.7 (50.2)	146.7 (42.5)
Low-density lipoprotein cholesterol, mean (SD), mg/dL	96.5 (38.6)	96.5 (38.6)	92.7 (34.8)
Serum creatinine, median (IQR), mg/dL	1.3 (1.0-1.9)	1.2 (0.9-1.5)	1.1 (0.9-1.8)
Estimated glomerular filtration rate, mean (SD), mL/min/1.73 m^2^	51.1 (27.6)	52.9 (27.1)	49.3 (28.2)
Comorbidity			
Diabetes	49 (40.8)	24 (40.0)	25 (41.7)
Chronic obstructive pulmonary disease	18 (15.0)	11 (18.3)	7 (11.7)
Chronic kidney disease	48 (40.0)	23 (38.3)	25 (41.7)
History of cerebrovascular accident	8 (6.7)	6 (10.0)	2 (3.3)
Medications			
Intravenous administration			
Vasoactive medications^d^	40 (33.3)	19 (31.7)	21 (35.0)
Loop diuretics	87 (72.5)	44 (73.3)	43 (71.7)
Recombinant human brain natriuretic peptide	6 (5.0)	2 (3.3)	4 (6.7)
Oral administration			
Mineralocorticoid receptor antagonists	57 (47.5)	27 (45.0)	30 (50.0)
β-Blockers	74 (61.7)	40 (66.7)	34 (56.7)
Angiotensin-converting enzyme inhibitors	8 (6.7)	3 (5.0)	5 (8.3)
Angiotensin receptor blockers	17 (14.2)	8 (13.3)	9 (15.0)
Angiotensin receptor-neprilysin inhibitor	38 (31.7)	15 (25.0)	23 (38.3)
Ivabradine	5 (4.2)	4 (6.7)	1 (1.7)
Calcium channel blockers	28 (23.3)	14 (23.3)	14 (23.3)
Statins	83 (69.2)	43 (71.7)	40 (66.7)
Digoxin	17 (14.2)	8 (13.3)	9 (15.0)
Antiplatelet mediation	74 (61.7)	37 (61.7)	37 (61.7)
Thrombin inhibitors	38 (31.7)	23 (38.3)	15 (25.0)
Antihyperglycemic medications	59 (49.2)	28 (46.7)	31 (51.7)
Lung function			
Maximal voluntary ventilation, median (IQR), L/min	24.0 (17.0-38.0)	24.5 (17.0-37.0)	24.0 (17.0-38.7)
FEV_1_:FVC, median (IQR)	0.7 (0.6-0.8)	0.7 (0.6-0.8)	0.7 (0.6-0.8)

Abbreviations: BMI, body mass index (calculated as weight in kilograms divided by height in meters squared); FEV_1_:FVC, ratio of forced expiratory volume in 1 second to forced vital capacity; LVEF, left ventricular ejection fraction; NYHA, New York Heart Association; Pao_2_:FIo_2_, ratio of arterial partial pressure of oxygen to fraction of inspired oxygen.

SI conversion factors: To convert troponin T to μg/L, multiply by 0.001; C-reactive protein to mg/L, multiply by 10; triglycerides to mmol/L, multiply by 0.0113; total and low-density lipoprotein cholesterol to mmol/L, multiply by 0.0259; and creatinine to μmol/L, multiply by 88.4.

^a^ Unless otherwise indicated, values are presented as number (percentage) of participants.

^b^ Regurgitation and stenosis of the mitral, tricuspid, and aortic valves.

^c^ After standard therapy, 2 patients improved from NYHA class IV at cardiac intensive care unit admission to NYHA class II at randomization.

^d^ Nitroglycerin, deslanoside, sodium nitroprusside, dopamine, and urapidil.

Key patient characteristics at CICU admission are shown in eTable 2 in Supplement 2, with physical therapy details presented in eTable 3 and eFigure 1 in Supplement 2. Overall, 39 patients (32.5%) had heart function classified as NYHA class III and 81 patients (67.5%) had class IV. Blood pressure was either greater than 150/100 mm Hg or less than 90/60 mm Hg in 50 patients (41.7%), and 57 (47.5%) required high-concentration oxygen to maintain Sao_2_ of 92% or higher, indicating hemodynamic instability and the need for standard therapy. Median duration of standard therapy was 39 hours (IQR, 24-70 hours) in the control group and 24 hours (IQR, 24-48 hours) in the intervention group. After stabilization, all patients were randomized and assessed for physical fitness. At baseline, 119 patients (99.2%) scored below level 4. Daily physical therapy was administered during the CICU stay (median, 5 sessions; IQR, 5-5 sessions). By study end, 54 of 60 control patients (90.0%) remained below level 4, while 53 of 60 intervention patients (88.3%) improved to above level 3. Median hospital stay was 10.5 days (IQR, 8.2-14.7 days) for the control group and 11.0 days (IQR, 8.0-16.0 days) for the intervention group. Median CICU stay was 6 days for both the control group (IQR, 5-8 days) and the intervention group (IQR, 5-7 days).

### Baseline Characteristics

At randomization, 49 (81.7%) control and 43 (71.7%) intervention patients had pulmonary crackles (Table 1). All received mechanical or noninvasive ventilation. Based on the NYHA classification, 58 (96.7%) control and 60 (100%) intervention patients had class III or IV HF. Additionally, 30 patients in the control group (50.0%) and 32 in the intervention group (53.3%) had a Pao_2_ to fraction of inspired oxygen ratio (FIo_2_) less than 300 mm Hg. At randomization, 19 patients in the control group (31.7%) and 21 in the intervention group (35.0%) received intravenous vasoactive medications, while 44 (73.3%) and 43 (71.7%), respectively, received intravenous loop diuretics. Maximal voluntary ventilation was 24.5 L/min (IQR, 17.0-37.0 L/min) in the control group and 24.0 L/min (IQR, 17.0-38.7 L/min) in the intervention group. No clinically significant differences were observed between the groups.

### Outcomes

**Figure 2.**
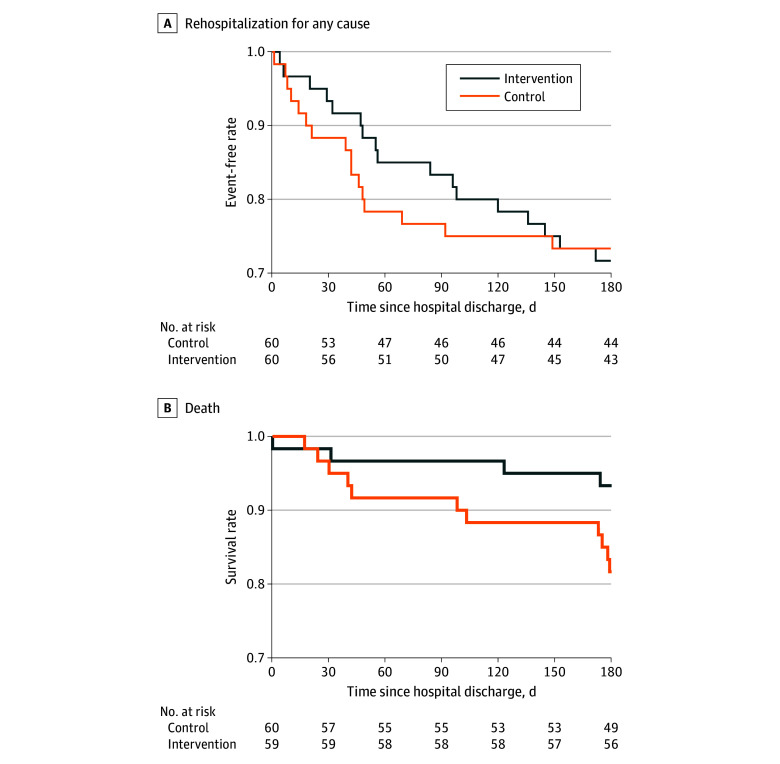
Rehospitalization and Survival During the Study

The primary and secondary outcomes are presented in Figure 2 and Table 2. At hospital discharge, the median SPPB score was 8.0 (IQR, 4.0-11.5) in the control group and 10.0 (IQR, 5.0-12.0) in the intervention group, with a median difference of 1.0 (95% CI, 0-2.0; *P* = .16). The 6-month all-cause rehospitalization rate was 26.7% in the control group (n = 16) and 28.3% in the intervention group (n = 17), with an unadjusted HR of 1.00 (95% CI, 0.51-1.99; *P* = .99) (Figure 2A).

**Table 2.** Intention-to-Treat Analysis Outcomes^a^

Outcome	Control group (n = 60)^b^	Intervention group (n = 60)^b^	Effect estimate (95% CI)	*P* value	Adjusted *P* value^c^
**Primary outcomes**					
SPPB score at hospital discharge^d^					
Overall	8.0 (4.0 to 11.5)	10.0 (5.0 to 12.0)	1.0 (0 to 2.0)^e^	.16^f^	.65
Balance	3.0 (1.5 to 4.0)	4.0 (2.0 to 4.0)	NA	NA	NA
Gait speed	2.0 (1.0 to 3.7)	3.0 (1.0 to 4.0)	NA	NA	NA
Chair rise	2.6 (1.0 to 4.0)	3.5 (1.0 to 4.0)	NA	NA	NA
6-mo All-cause rehospitalization	16 (26.6)	17 (28.3)	1.00 (0.51 to 1.99)^g^	.99	>.99
**Secondary outcomes**					
6-mo Mortality^h^	11 (18.3)	4 (6.6)	0.35 (0.11 to 1.09)^g^	.07	.34
ADL score at CICU discharge					
Baseline	40.0 (35.0 to 45.0)	42.5 (35.0 to 50.0)	NA	NA	NA
Final	77.0 (50.0 to 90.0)	90.0 (63.0 to 100)	4.99 (−4.99 to 14.99)^i^	.34^j^	>.99
PCS score from SF-36 at 6-mo after hospital discharge^k^					
Baseline	38.5 (28.8 to 51.0)	45.5 (30.2 to 56.5)	NA	NA	NA
Final	55.7 (29.4 to 73.2)	57.8 (34.2 to 72.2)	1.28 (−8.00 to 11.54)^i^	.99^j^	>.99
**Exploratory outcome**					
PERME score at CICU discharge					
Baseline	12.0 (10.0 to 18.0)	14.0 (11.0 to 16.5)	NA	NA	NA
Final	19.5 (15.0 to 24.3)	25.0 (19.2 to 28.0)	2.76 (0.77 to 4.74)^l^	.01^m^	.04

Abbreviations: ADL, activities of daily living; CICU, cardiac intensive care unit; NA, not applicable; PCS, Physical Component Summary; PERME, Perme Intensive Care Unit Mobility; SF-36, 36-Item Short Form Survey; SPPB, Short Physical Performance Battery.

^a^ Each group experienced 2 instances of missing data due to interrupted trials involving SPPB, PERME, and SF-36 assessments. Descriptions of scores are given in the Outcomes subsection of the Methods section.

^b^ Data are presented as number (percentage) of participants for all-cause rehospitalization and 6-month mortality and as median (IQR) for all other outcomes.

^c^ Calculated using the Holm-Bonferroni method.

^d^ Baseline scores were not recorded for participants unable to walk or rise from a chair. One patient who died after the intervention but before hospital discharge had their SPPB score recorded as 0.

^e^ Median difference (95% CI), estimated using the Hodges-Lehmann method.

^f^ Calculated using the Mann-Whitney *U* test.

^g^ Unadjusted hazard ratio (95% CI), estimated using Cox proportional hazards regression analysis.

^h^ Deaths included 1 patient who died before hospital discharge.

^i^ Median difference (95% CI) between baseline and final scores.

^j^ Calculated using the Wilcoxon rank-sum test.

^k^ For patients who died, the PCS score was recorded as 0.

^l^ Median difference (95% CI), calculated using analysis of covariance.

^m^ Calculated using analysis of covariance

The 6-month mortality rate was 18.3% for the control group (n = 11) and 6.7% for the intervention group (n = 4), with an unadjusted HR of 0.35 (95% CI, 0.11-1.09; adjusted *P* = .34) (Figure 2B). The median difference in ADL scores between the 2 groups was 4.99 (95% CI, −4.99 to 14.99; adjusted *P* > .99) after adjusting for baseline levels. The health-related quality-of-life measure of the PCS from the SF-36 score showed a difference of 1.28 (95% CI, −8.00 to 11.54; adjusted *P* > .99) at 6 months after hospital discharge. The PERME score at CICU discharge was 19.5 (IQR, 15.0-24.3) for the control group and 25.0 (IQR, 19.2-28.0) for the intervention group, with a median difference of 2.76 (95% CI, 0.77-4.74; adjusted *P* = .04) after adjusting for baseline levels. Cardiopulmonary function was assessed in both groups at CICU discharge, and the differences between the groups were minimal (eTable 4 in Supplement 2). PERME score benefits were consistent across subgroups, including by sex, smoking status, Pao_2_:FIo_2_, and ischemic cardiomyopathy status (eTable 5 in Supplement 2).

## Discussion

Our findings suggest that short-term stabilization therapy for patients with severe ADHF during the acute phase allows the initiation of the AHF-CR intervention, even in the critical illness stage in the CICU. Compared with patients receiving usual care, those receiving AHF-CR showed no significant improvement in SPPB scores at hospital discharge, and the intervention did not affect rehospitalization or mortality within 6 months. However, AHF-CR improved PERME scores. These findings suggest that AHF-CR is feasible for critically ill patients with ADHF, does not increase rehospitalization or mortality, and may enhance physical function (eFigure 2 in Supplement 2).

In this study, the mean age of participants was 68.6 years. After short-term standard therapy in the CICU, nearly all participants had severely impaired heart function, classified as NYHA class III or IV, and required mechanical or noninvasive ventilation to maintain normal Sao_2_ levels. A total of 72.5% of patients continued to present with pulmonary edema requiring intravenous diuretics; 51.7% had a low Pao_2_:FIo_2_, and 33.3% were still receiving vasoactive medications. These characteristics align with previous studies of patients with ADHF in the CICU.^30,31^ Some studies have focused on early CR for ICU patients with critical illness, who often have a low Pao_2_:FIo_2_ and require mechanical or noninvasive ventilation.^18,19^ In earlier reports on early CR for patients with ADHF, patients typically received standard anti-HF medications, including loop diuretics or β-blockers, and had a baseline 6-minute walk distance of about 200 m, suggesting that they were recovering inpatients.^12,32,33^ Thus, the patients in our study, in the critical illness stage, differ markedly from patients in these prior reports.

Given that hemodynamic instability contraindicates exercise therapy,^5,6^ clinical guidelines for treating critically ill patients with ADHF are limited. In this study, we defined patient stability parameters. Despite ongoing therapies to control edema and stabilize blood pressure, our data suggest that initiating AHF-CR is feasible, with only 2 patients discontinuing in the intervention group, similar to the control group. The incidence of adverse events during early mobilization of ICU patients receiving mechanical ventilation is relatively low, although cardiovascular effects, such as unstable blood pressure, reduced oxygen saturation, and increased heart rate, are the major observed adverse events.^16,34^ No increase in mortality or rehospitalization was observed, and cardiopulmonary function did not differ significantly between groups, supporting the safety of AHF-CR.

The PERME score, an ICU-specific tool for assessing mobility, significantly improved after AHF-CR. Similarly, Schweickert et al^16^ showed that early physical and occupational therapy in ICU patients (excluding those with ADHF) accelerated key mobility milestones, such as getting out of bed and walking. Although exploratory, our findings suggest that early CR may enhance mobility in critically ill patients. The REHAB-HF (Rehabilitation Therapy in Older Acute Heart Failure Patients) trial^12^ focused on older patients with frailty, ADHF, and lower baseline function (mean SPPB score of approximately 6 compared with 8.0 in the control group in our study). The cohort in our study had more severe illness, with median N-terminal pro–B-type natriuretic peptide levels and NYHA class IV rates roughly twice those of patients in the REHAB-HF trial. While the REHAB-HF trial demonstrated a significant 1.5-point SPPB improvement with long-term CR in 349 patients, our trial observed a nonsignificant 1-point gain with early AHF-CR. These studies addressed different aspects (long-term efficacy vs early feasibility), highlighting that early initiation and continuation of CR may offer greater benefits for patients with ADHF.

### Limitations

This study has several limitations. First, the improvement in PERME scores was based on exploratory analysis, and more robust evidence is needed to confirm the mobility benefits of short-term CR in critically ill patients with ADHF. Second, although early physical and occupational therapy may reduce long-term cognitive impairment in ICU patients,^35^ the long-term impact of AHF-CR remains unclear. Third, the single-center design and exclusively Chinese cohort may limit generalizability. Fourth, this was a single-blind trial; while outcome assessors were blinded, patients were not. Fifth, although the control group received usual care, including early mobility and exercise therapy, we did not quantify their mobility, which may have diminished the observed effect. Sixth, no separate power analysis was performed for the coprimary outcome of all-cause rehospitalization, potentially limiting statistical power; however, the observed HR (1.00 [95% CI, 0.51-1.99]) suggests only a negligible effect size.

## Conclusions

In this randomized clinical trial of critically ill patients with ADHF, initiating AHF-CR after a brief stabilization period was feasible. Although the AHF-CR program did not lead to significant improvements in SPPB scores or rehospitalization rates, it may be associated with enhanced mobility status and some physical benefits.

## Notes

Supplement 1.
Trial Protocol

Supplement 2. **eFigure 1.** Length of Stay for Patients Before and After Randomization **eFigure 2.** Summary of the Study **eTable 1.** Assessment, Protocol, and Goals of the Tailored Cardiac Rehabilitation Program (AHF-CR) **eTable 2.** Key Characteristics of Patients at CICU Administration **eTable 3.** Characteristics of Physical Therapy During CICU Stay **eTable 4.** Supporting Outcomes About Cardiopulmonary Function at CICU Discharge **eTable 5.** Subgroup Analysis of the PERME Score

Supplement 3.
Data Sharing Statement
