# Supplementary material for: Early Cardiac Rehabilitation for Critically Ill Patients With Acute Decompensated Heart Failure: A Randomized Clinical Trial
Source: JAMA Netw Open. 2025 Jul 30;8(7):e2524141. doi: 10.1001/jamanetworkopen.2025.24141 (PMC12311714; doi:10.1001/jamanetworkopen.2025.24141)
Supplement: Supplement 1. — Trial Protocol [file jamanetwopen-e2524141-s001.pdf]

# Clinical Study Protocol

**Project Title:** **Efficacy and Safety of Phase I Cardiac Rehabilitation in Patients with Acute Decompensated Heart Failure**

**Principal Investigator:** **Linjing Wu**

**Institution:** **Xiamen Cardiovascular Hospital, Xiamen University**

**Email:** **3620883@qq.com**

## Declaration of Integrity

Our research team solemnly declares that all operations in this study are conducted strictly according to the research protocol, and all trial data are recorded accurately and truthfully. The related research outcomes, including technical standards, patents, and other intellectual properties, belong to our research team. The team is fully aware of the legal responsibilities associated with this declaration.

**Study Title**

Efficacy and Safety of Phase I Cardiac Rehabilitation in Patients with Acute Decompensated Heart Failure

**Protocol Version Number**

1.1

**Funding Source:**

Self-funded

**Study Timeline and Major Research Tasks**

| Period | Major Research Tasks | Goals |
|---|---|---|
| Jan 2021 - Mar 2021 | Refine the research plan, submit the research plan to the Ethics Committee for review | Complete research plan and ethical review |
| Mar 2021 - Sep 2021 | Subject recruitment, conduct research according to the project design | Complete clinical research |
| Oct 2021 - Mar 2022 | Subject recruitment, conduct research according to the project design | Complete clinical research |
| Mar 2022 - Sep 2022 | Organize data | Complete paper writing and publication |

# Contents

1. Background ........ 5
2. Inclusion Criteria ........ 9
3. Exclusion Criteria ........ 10
4. Randomization and Sample Size Calculation ........ 10
5. Enrollment and Baseline Treatment Collection ........ 11
6. Intervention ........ 11
7. Evaluation indicators ........ 15
8. Definition of Efficacy Recognition for Participants ........ 15
9. Definition, Identification Methods, and Management System for Adverse Events and Reactions ........ 16
10. Ethical Considerations ........ 17
11. Participant Recruitment ........ 18
12. Collection of General Information of Participants ........ 18
13. Standard Operating Procedures ........ 19
14. Statistical Methods ........ 19
15. Participant Management ........ 19
16. Specimen Management ........ 19
17. Drug and Equipment Management ........ 20
18. Data Management ........ 20
19. Composition and Responsibilities of the Data Safety and Monitoring Committee ........ 20
20. Research Team ........ 20
21. Intellectual Property ........ 20
22. Publication Plan ........ 20
23. Original Data Sharing Plan ........ 20
24. Post-Trial Treatment and Management for Participants ........ 21

## 1. Background

### 1） Heart Failure Predominantly in Elderly Patients with Poor Prognosis, High Rehospitalization, and Mortality Rates

Heart failure (HF) is the severe stage of various cardiovascular diseases, with high morbidity and mortality rates becoming a global public health issue affecting the elderly population[1]. In China, over three-quarters of heart failure patients are middle-aged and elderly, and this number continues to increase each year [2]. Acute decompensated heart failure (ADHF), marked by sudden worsening or changes in heart failure symptoms, is the leading cause of hospitalization and cardiac death among heart failure patients [3]. It is also a major reason for hospitalization in patients over 65 years old. During hospitalization, ADHF patients often experience reduced physical endurance due to prolonged bed rest. They are prone to complications such as infections, pressure injuries, deep vein thrombosis, and acquired muscle weakness, which can lead to functional decline, frailty, rehospitalization, and significantly reduced quality of life [5]. Patients with ADHF have a poor prognosis. Studies indicate that hospitalization costs for ADHF patients account for 75% of the total heart failure treatment costs abroad, with an in-hospital mortality rate of 7% and a rehospitalization rate of 30%. In China, the in-hospital mortality rate is 4.1%, with a rehospitalization rate of approximately 50% within six months, and a five-year mortality rate as high as 60% [2, 6-7,10].

### 2） Early Exercise Rehabilitation Improves Heart Failure Patients' Outcomes but Evidence for ADHF Patients is Limited

Cardiac rehabilitation is now recognized as a crucial adjunctive treatment for heart failure (HF) patients. Recent guidelines recommend that HF patients engage in safe and effective exercise training (or regular physical activity) early on to enhance their functional status, with a Class Ia recommendation. [8-10]. Moreover, related guidelines and expert consensus [9-12] suggest that early exercise training can improve exercise endurance, enhance health-related quality of life, and reduce rehospitalization rates in HF patients. For severe HF patients, personalized early activities should be encouraged as soon as the underlying cause is controlled and standard medication treatment is administered to prevent further disability. However, due to safety concerns, ADHF

patients are often excluded from studies involving HF patients. This exclusion has led to limited research on the safety and clinical effects of early exercise rehabilitation in ADHF patients, resulting in its absence from guideline recommendations. In 2017, Reeves *et al*. [13] conducted a preliminary study on 27 hospitalized ADHF patients, implementing early functional exercises including balance, strength, flexibility, and endurance rehabilitation, lasting until 12 weeks post-discharge. The results showed improved physical function at three months and a decreased rehospitalization rate at six months. Similarly, Oliveira *et al*. [14] explored the combination of exercise training and non-invasive ventilation in hospitalized ADHF patients, finding that exercise training was safe with no adverse events, improved the six-minute walk test (6MWT), and reduced dyspnea and hospitalization duration. However, like Reeves et al., this study had a small sample size and weak evidence strength, indicating the need for larger randomized controlled trials to validate the effectiveness of early activities in ADHF patients. In 2019, Motoki H. et al. [15] studied 171 ADHF patients undergoing a two-phase inpatient rehabilitation program, including resistance training and aerobic exercise. The study found improved Barthel Index (BI) scores post-rehabilitation and a significant association between the ability to perform daily activities and mortality rates, with BI being an independent predictor of death. However, the retrospective nature of this study introduced selection bias and varied time distribution, highlighting the need for further prospective, large-scale studies to confirm these findings. Motoki H. et al., Suzuki et al. [16] conducted a retrospective cohort study across over 100 hospitals involving 8,351 ADHF patients hospitalized between 2014 and 2017. They observed that the non-rehabilitation group experienced a greater decline in the Barthel Index compared to the early rehabilitation group (18.7% vs. 12.4%). Multivariate linear regression analysis showed a significant negative correlation between early rehabilitation and decline in the Barthel Index. Conversely, Delgado et al. [17] found that an early aerobic exercise program did not improve the Barthel Index in ADHF patients. Domestic research also explored early rehabilitation effects in acute heart failure patients. Wang Zhongjie et al. [18] randomly divided 60 severe heart failure patients into experimental and control groups. The experimental group received a three-phase early activity intervention, resulting in lower incidences of pulmonary infection, electrolyte disorders, constipation, embolism, or pressure sores compared to the control group. However, the study's small sample size, non-standardized exercise prescriptions, and lack of rigorous design limit its generalizability. In 2020, Wang Hong et al. [19] implemented a four-step

early rehabilitation program for 200 acute heart failure patients. The experimental group showed better outcomes in quality of life, the six-minute walk test (6MWT), and forced expiratory volume in one second (FEV1) compared to the control group. While the individualized, progressive activity strategy demonstrated good safety and efficacy, the study focused on patients with left ventricular ejection fraction (LVEF) > 40%, leaving the applicability and effectiveness for ADHF patients with LVEF ≤ 40% unclear.

**However, these studies typically have small sample sizes and limited prospective research. There is no consensus on the optimal timing, intensity, form, or duration of early exercise rehabilitation. Safety indicators mainly focus on complications, with a lack of predictive tools for the safety of early activities. Therefore, there is a need to develop a scientific, systematic, safe, and effective exercise training program for these patients.**

**This study aims to develop a Phase I cardiac rehabilitation program for intensive ADHF patients and evaluate its efficacy and safety by assessing physical fitness, mobility, quality of life, and heart and lung function.**

**References**

[1] Go A S, Mozaffarian D, Roger V L, et al. Executive summary: heart disease and stroke statistics—2013 update: a report from the American Heart Association[J]. Circulation, 2013, 127(1): 143-152.

[2] 王华,李莹莹,柴坷,等.中国住院心力衰竭患者流行病学及治疗现状[J].中华心血管病杂志,2019,47(11):865-874. .

[3]Kurmani S, Squire I. Acute heart failure: definition, classification and epidemiology[J]. Current heart failure reports, 2017, 14(5): 385-392.

[4] Cheng R K, Cox M, Neely M L, et al. Outcomes in patients with heart failure with preserved, borderline, and reduced ejection fraction in the Medicare population[J]. American heart journal, 2014, 168(5): 721-730. e3.

[5]Murad K, Kitzman D W. Frailty and multiple comorbidities in the elderly patient with heart failure: implications for management[J]. Heart failure reviews, 2012, 17(4-5): 581-588.

[6] Farmakis D, Parissis J, Lekakis J, et al. Acute heart failure: epidemiology, risk factors, and prevention[J]. Revista Española de Cardiología (English Edition), 2015, 68(3): 245-248.

[7]代培胜,韩亚岩,崔娟敏,等.重组人 B 型脑利钠钛治疗慢性心力衰竭急性发作失代偿期

的临床疗效观察[J].医学综述,2014,20(2):719-722.

[8] Yancy C W, Jessup M, Bozkurt B, et al. 2013 ACCF/AHA guideline for the management of heart failure: a report of the American College of Cardiology Foundation/American Heart Association Task Force on Practice Guidelines[J]. Journal of the American College of Cardiology, 2013, 62(16): e147-e239.

[9] Ponikowski P, Voors A A, Anker S D, et al. 2016 ESC Guidelines for the diagnosis and treatment of acute and chronic heart failure: The Task Force for the diagnosis and treatment of acute and chronic heart failure of the European Society of Cardiology (ESC) Developed with the special contribution of the Heart Failure Association (HFA) of the ESC[J]. European heart journal, 2016, 37(27): 2129-2200.

[10]中华医学会心血管病学分会心力衰竭学组,中国医师协会心力衰竭专业委员会,中华心血管病杂志编辑委员会.中国心力衰竭诊断和治疗指南 2018[J].中华心力衰竭和心肌病杂志（中英文）,2018,2(4):196-225..

[11]Flynn K E, Piña I L, Whellan D J, et al. Effects of exercise training on health status in patients with chronic heart failure: HF-ACTION randomized controlled trial[J]. Jama, 2009, 301(14): 1451-1459.

[12] Piepoli M F, Conraads V, Corra U, et al. Exercise training in heart failure: from theory to practice. A consensus document of the Heart Failure Association and the European Association for Cardiovascular Prevention and Rehabilitation[J]. European journal of heart failure, 2011, 13(4): 347-357.

[13] Reeves G R, Whellan D J, O'Connor C M, et al. A novel rehabilitation intervention for older patients with acute decompensated heart failure: the REHAB-HF pilot study[J]. JACC: Heart Failure, 2017, 5(5): 359-366.

[14] Oliveira M F, Santos R C, Artz S A, et al. Safety and efficacy of aerobic exercise training associated to non-invasive ventilation in patients with acute heart failure[J]. Arquivos brasileiros de cardiologia, 2018, 110(5): 467-475.

[15]Motoki H, Nishimura M, Kanai M, et al. Impact of inpatient cardiac rehabilitation on Barthel Index score and prognosis in patients with acute decompensated heart failure[J]. International journal of cardiology, 2019, 293: 125-130.

[16]Suzuki S, Momosaki R, Watanabe T, et al. Effectiveness of Early Rehabilitation for Acute

Heart Failure: A RETROSPECTIVE COHORT STUDY[J]. Journal of cardiopulmonary rehabilitation and prevention, 2019, 39(4): E23-E25.

[17] Delgado B M, Lopes I, Gomes B, et al. Early rehabilitation in cardiology–heart failure: The ERIC-HF protocol, a novel intervention to decompensated heart failure patients rehabilitation[J]. European Journal of Cardiovascular Nursing, 2020: 1474515120913806.

[18]王中洁,严华,黄春燕. 重度心力衰竭患者早期活动的探讨[J]. 中国实用护理杂志,2006,07:14-16.

[19]王红,程青,李迎新,张茜茜,许红凤. 急性心力衰竭代偿期患者早期康复活动方案的构建及应用效果[J]. 中华护理杂志,2020,04:524-528.

**2. Inclusion Criteria**

（1）Age ≥ 18 years old；

（2）Acute decompensated heart failure was defined according to the “2021 ESC Guidelines for the diagnosis and treatment of acute and chronic heart failure”. Combined with symptoms, signs and laboratory results, and meet all the following conditions:

(2.1) At least one symptom was worsened from baseline: (a) dyspnea (dyspnea after exercising, paroxysmal nocturnal dyspnea or orthopnea), (b) venous congestion of systemic circulation (edema of lower extremities, liver congestion, ascites), and (c) tissue hypo-perfusion (oliguria or anuria, cold clammy limbs, consciousness disorder, hyper-lacticacidemia, or metabolic acidosis);

(2.2) At least one of the following signs of heart failure: (a) pulmonary edema on exam or by chest X ray, (b) Increased b-type natriuretic peptide or N-terminal prohormone BNP, and (c) abnormal cardiac structure and/or function by cardiac ultrasound;

（3）Patients who have New York Heart Association Cardiac Function class III or IV symptoms at admission.

（4）Clinical stability has been achieved for 24 h after optimizing treatment;

（5）Patient was independent with basic activities of daily living including the ability to ambulate independently (with or without the use of an assistive device) prior to admission.

### 3. Exclusion Criteria

(1) Acute coronary syndrome within 2 days; onset of chest pain within 8 h; or combined with mechanical complications;

(2) Fatal arrhythmia, high-grade atrioventricular block, new onset atrial fibrillation or atrial flutter;

(3) Severe aortic valve stenosis;

(4) Acute myocarditis, acute pericarditis, or acute infective endocarditis;

(5) Severe hypertrophic obstructive cardiomyopathy;

(6) Patients with chronic kidney disease stage 4 and 5 undergoing long-term hemodialysis or requiring long-term hemodialysis within the next 6 months;

(7) A recent embolism, atrium, or ventricular thrombosis, thrombophlebitis;

(8) Patients who require a pacemaker, implantable cardioverter defibrillator (ICD), or ventricular assist device, or those awaiting cardiac transplantation within the next 6 months;

(9) Those who had received standardized cardiac rehabilitation within the past 6 months;

(10) Severe cognitive impairment (MMSE ≤ 9 points);

(11) Inability or unwillingness to comply with the study requirements;

(12) Patients who dropped out or discharged during the study period.

### 4. Randomization and Sample Size Calculation

Subjects who meet the inclusion and exclusion criteria and are ultimately enrolled will be assigned to the control group and intervention group by random number methods. ① Random number sequences will be generated through a randomizer website by personnel not involved in the study to ensure impartiality. The random numbers will be sealed, opaque envelopes and securely stored. ② After enrollment, researchers will contact a random number custodian via phone to determine the group assignments. The custodian will sequentially open the envelopes to reveal the group assignments. Subjects with odd random numbers will be assigned to the experimental group, while those with even random numbers will be assigned to the control group.

According to the results of the previous study, the baseline SPPB sores was 6.0 ± 2.8 in CR group and 6.1 ± 2.6 in control group, the SPPB sores at 3 months was 8.3 ± 0.2 in CR group and 6.9

± 0.2 in control group. To minimize both false positive and false negative errors, the type-I error rate (a) is set at 0.01 instead of 0.05 and the type-II error rate is set at 0.1 instead of 0.2. The sample size is calculated according to the following formula: n =2s2 × f (a,b)/(μ1-μ2)2. The sample size of each group was estimated to be 44 per group. Taking approximate a 20% drop-off rate and a 7% missing rate into account, the final sample size was set to 60 per group. Therefore, a total of 120 subjects will be recruited in this study.

## 5. Enrollment and Baseline Treatment Collection

(1) Upon signing and dating the informed consent form, the patient officially becomes a participant in this sub-study. The date of consent must be documented in the patient's medical record. Following enrollment, a baseline assessment will be conducted to confirm that the inclusion and exclusion criteria are met (refer to the inclusion registration form).

(2) Baseline Data Collection

① Demographic Data: Includes name, gender, age, date of birth, height, weight, etc.

② Medical History: Includes diagnosis, comorbidities, medication treatments, and heart function classification.

③ Admission Tests: Includes complete blood count, biochemical function tests, cardiac injury markers, NT-proBNP, glycated hemoglobin, electrocardiogram (ECG), echocardiogram, and lower limb vascular ultrasound.

④ Daily Activity Assessment: Includes smoking status, alcohol consumption, and Barthel Index (BI).

## 6. Intervention

All ADHF patients in the CICU will undergo standard therapy to stabilize their condition before starting the intervention. The intervention will begin once the following conditions are met.

**A.** No onset or recurrence of chest pain in the past 8 hours;

**B.** No new symptoms of decompensated heart failure;

**C.** No new arrhythmias or dynamic changes on the electrocardiogram within the past 8 hours;

**D.** No further elevation in troponin levels;

**E.** No increases in vasoactive drug doses;

**F.** A resting heart rate of <110 beats per minute;

**G.** Resting blood pressure between 90–150/60–100 mmhg;

**H.** Blood oxygen saturation (sao₂) ≥92%.

**1) Control Group:**

Patients in the control group will adhere to the recommended scheme outlined in the "2021 ESC Guidelines for the Diagnosis and Treatment of Acute and Chronic Heart Failure," implemented by the responsible nurse who will guide the patients through exercises. Specific measures include:

**Decompensated Phase:** The primary approach is bed rest. If there are no contraindications, standard pneumatic therapy will be performed to prevent deep vein thrombosis.

**Post-Improvement Phase:** After the clinical condition improves and without causing symptoms, the responsible nurse will guide patients through progressively more active movements. This includes passive and active limb exercises, diaphragmatic breathing, pursed-lip breathing exercises, and gradually transitioning to out-of-bed activities.

**2) Intervention Group:**

The cardiac rehabilitation program, based on impedance cardiography findings, will be implemented by the " Rehabilitation Team" nurses. Before rehabilitation, the experimental group will undergo a safety assessment using the impedance cardiography combined with a leg lift load test. Patients who test positive in the leg lift test will begin the early 7-level rehabilitation program including resistance training, treadmill training, working and others (Table 1). Depending on the patient's consciousness, heart function classification, and limb strength, different levels of exercise categories will be selected. Based on the physical fitness assessment, a personalized exercise training intensity will be formulated. During exercise, the patient's heart rate response and Borg rating will determine whether they can progress to the next stage.

**Table 1: Early 7-Level Rehabilitation Program for ADHF Patients**

| | **Level** | **1** | **2** | **3** | **4** | **5** | **6** | **7** |
|---|---|---|---|---|---|---|---|---|
| **Assessment** | **Unconscious** | Unconscious | Conscious | Conscious | Conscious | Conscious | Conscious | Conscious |

| | | | | | | | | |
|---|---|---|---|---|---|---|---|---|
| | **Muscle strength** | Upper limb muscle strength < 3 level | Upper limb muscle strength ≥3 level | Limb muscle strength ≥ 3 level | Limb muscle strength ≥ 4 level | Limb muscle strength ≥4 level | Lower limbs muscle strength 5 level | Lower limbs muscle strength 5 level |
| | **NYHA classification III** | III–IV | III–IV | III | II-III | II–III | II–III | II–III |
| **Rehabilitation plans and programs** | **Range of Motion (ROM)** | Passive ROM once per day | Active/passive ROM once per day | Active ROM once per day | Active ROM once per day | Active ROM once per day | Active ROM once per day | Active ROM once per day |
| | **Treadmill training** | Passive bed treadmill training for 10–20min | Passive bed treadmill training for 10–20min | Bed treadmill training for 10–20min | Bed treadmill training for 10–20min | Bed treadmill training for 10–20min | Bed treadmill training for 10–20min | Bed treadmill training for 10–20min |
| | **Breathing Training** | / | / | Progressive breathing muscle training | Progressive breathing muscle training | Progressive breathing muscle training | Progressive breathing muscle training | Progressive breathing muscle training |
| | **Resistance Training** | / | / | Progressive resistance training 15 times per group (elbow flexion and extension movements), 3 groups, rest for 2min between each group | Progressive resistance training 15 times per group (elbow flexion and extension movements for 3 groups and abdominal crunch for 1 group and hip bridge for 1 group), rest for 2min between each group | Progressive resistance training 15 times per group (elbow flexion and extension movements for 3 groups and abdominal crunch for 1 group and hip bridge for 1 group), rest for 2min between each group | Progressive resistance training 15 times per group (elbow flexion and extension movements for 3 groups and abdominal crunch for 1 group and hip bridge for 1 group), rest for 2min between each group | Progressive resistance training 15 times per group (elbow flexion and extension movements for 3 groups and abdominal crunch for 1 group and hip bridge for 1 group), rest for 2min between each group |

| | | | | | | | | |
|---|---|---|---|---|---|---|---|---|
| | **Sitting in bed (bed head elevation >45 ◦)** | / | Sitting in bed 5min, 2 times per day | Sitting in bed 5min, 2 times per day | Sitting in bed 5min, 2 times per day | Sitting in bed 5min, 2 times per day | Sitting in bed 5min, 2 times per day | Sitting in bed 5min, 2 times per day |
| | **Sitting at the edge of the bed** | / | / | Sitting at the edge of the bed 5min, 2 times per day | Sitting at the edge of the bed 5min, 2 times per day | Sitting at the edge of the bed 5min, 2 times per day | Sitting at the edge of the bed 5min, 2 times per day | Sitting at the edge of the bed 5min, 2 times per day |
| | **Standing and stepping** | / | / | / | Standing/stepping for 2 min | Standing/stepping for 2 min | Standing/stepping for 5 min | Standing/stepping for 5 min |
| | **Bed to chair transfer** | / | / | / | Sitting in chair 5min per day, 2 times per day | Sitting in chair 5min per day, 2 times per day | Sitting in chair 5min per day, 2 times per day | Sitting in chair 5min per day, 2 times per day |
| | **Walking bedside bed** | / | / | / | / | Walking training by supported by walker for 2min | Walking training by supported by walker for 2min | Walking training by supported by walker for 2min |
| **Goals** | **Heart rate response (compared with resting heart rate)** | Increase 5–15 beats per minute | Increase 5–15 beats per minute | Increase 20–30 beats per minute | Increase 20–30 beats per minute | Increase 20–30 beats per minute | Increase 20–30 beats per minute | Increase 20–30 beats per minute |
| | **Borg Score** | / | <12 | 12-13 | 12-13 | 12–13 | 12–13 | 12–13 |
| | **Perme Score** | / | Sitting up on bed for a score of 3 | Meditation balance for a score of 3 | Sit-to-stand transfer or standing balance for a score of 3 ing balance 3 points | Bed to chair transfer for a score of 3 | Walking training for a score of 3 | / |

## 3) Exercise Pause Indicators:

①New arrhythmias on ECG monitoring, heart rate increase ≥ 20 beats/min, dynamic changes

in the ST segment on the ECG. ②Blood pressure does not increase but decreases, or systolic blood pressure increases by more than 40 mmHg. ③Blood oxygen saturation < 92%. ④Symptoms of exercise intolerance, such as chest tightness, shortness of breath, palpitations, or difficulty breathing.

**7. Evaluation indicators**

**(1) Primary outcomes**

Short Physical Performance Battery (SPBB) score at discharge to evaluate the efficacy of early exercise rehabilitation.

Rehospitalization and mortality at 6 month to evaluate the safety of early exercise rehabilitation.

**(2) Secondary outcomes**

Evaluation of exercise capacity and daily living abilities: activities of daily living (ADL) at hospital discharge, and the Short-Form 36-Item Health Survey (SF-36) at 6-month post-discharge.

In-hospital mortality rate and 6-month all-cause mortality rate.

**(3) Other outcomes**

Cardiopulmonary function at hospital discharge was evaluated based on echocardiographic findings, laboratory results, and impedance cardiography, including myocardial necrosis markers, NT-proBNP, echocardiography, lower limb vascular ultrasound, left ventricular ejection time (LVET), heart rate (HR), stroke volume, cardiac output, cardiac index, cardiac contractility index, early diastolic filling rate, left heart work index, systemic vascular resistance index, ventricular systolic and diastolic waves, and pulmonary function indicators (e.g., FEV1, MVV).

**8. Definition of Efficacy Recognition for Participants**

**(1)** Participants have the right to withdraw from the clinical study at any stage. Researchers are also obligated to take necessary measures, including proactively deciding to withdraw participants from the clinical study, to ensure participants' safety and rights.

**(2)** Researchers should proactively consider withdrawing participants from the clinical study under the following circumstances:

1） The participant's condition worsens.

2） Serious adverse complications related to the study intervention occur.

3） Other situations that may increase the participant's risk or compromise the reliability of the study results.

**(3)** Voluntary Withdrawal by Participants:

1） Participants should not face any discrimination or retaliation due to their withdrawal, and their medical treatment and rights should not be affected.

2） Researchers should endeavor to understand the reasons for participants' voluntary withdrawal from the clinical study and record the related information in the original documents.

**(4)** If participants withdraw from the study due to any adverse events, researchers should follow up according to the protocol or guide the resolution of the adverse events and record the follow-up information in the original documents for archival purposes.

**(5)** Information related to participants' withdrawal from the clinical study should be documented in the original files.

**(6)** Upon learning of or deciding on participants' withdrawal from the clinical study, researchers should complete all feasible evaluation items and data collection

**(7)** Participants' withdrawal from the study does not imply that the data already obtained from them should be excluded from the clinical study. The data collected up to the point of withdrawal should be retained as part of the study database and should not be deleted or ignored.

## 9. Definition, Identification Methods, and Management System for Adverse Events and Reactions

**(1)** Adverse events refer to any unfavorable medical occurrences in patients or clinical research participants during the study intervention, which may not necessarily have a causal relationship with the treatment intervention. Adverse events can include any unfavorable and unexpected signs, symptoms, or diseases temporally associated with the study intervention, regardless of whether they are related to the study intervention.

**(2)** Serious adverse events refer to any unfavorable events occurring at any time during the observation period, including events that result in extended hospitalization, disability, impact on work capacity, life-threatening conditions, or death.

**(3)** Upon confirming an adverse event, an initial determination should be made on whether it qualifies as a serious adverse event. Ordinary adverse events should be managed clinically according to the actual situation, and the adverse event should be recorded in the CRF (case report form).

**(4)** Management of Serious Adverse Events:

1） If a serious adverse event threatens the life of the participant or patient, immediate and adequate medical intervention should be provided to ensure participant safety and alleviate any conflict.

2） The central principal investigator and the ethics committee of the research unit must be notified within 12 hours of first learning about the serious adverse event.

3） The study leader must be informed within 24 hours.

4） Proper communication and follow-up work should be conducted with the participant and their family.

**(5)** This observational study does not impose intervention measures on patients, and therefore, there are no compensation measures associated with the occurrence of adverse events.

## 10. Ethical Considerations

Ethics Committee: Xiamen Cardiovascular Hospital, Xiamen University

Approval Process: Complete the ethics approval application form and attach relevant approval materials.

Informed Consent: Before enrolling each patient in this study, the research physician is responsible for providing a complete and comprehensive explanation of the study's purpose and potential risks to the patient. Patients should be informed about their rights, the risks they undertake, and the benefits. During the research process, the research physician ensures the confidentiality of each patient's identity, health data, and related information. If the research results need to be used in scientific reports or published articles, the confidentiality of the patient’s identity, health data, and

family-related information must be maintained and not used for commercial purposes. Patients should sign the informed consent form before enrollment, which will be retained in the CRF.

Ethical Norms: This clinical study must adhere to the Declaration of Helsinki (2000 version). The study can only begin after receiving approval from the hospital's ethics committee. Any modifications to the study protocol during the clinical study must be reported to and recorded by the ethics committee.

Registration Agency: Xiamen Cardiovascular Hospital, Xiamen University

## 11. Participant Recruitment

Recruitment Location: CICU, Xiamen Cardiovascular Hospital, Xiamen University

Recruitment Method: Open recruitment

Screening Process: Screening according to the inclusion and exclusion criteria

Screening Personnel: Cuilian Dai, Linjing Wu

## 12. Collection of General Information of Participants

Data Collection Personnel: Fanqi Meng, Wei Yan, Jiahua Li

**Content of General Information:**

（1） Demographic Data: Name, gender, age, date of birth, height, weight, etc.

（2） Biological History: Diagnosis, comorbidities, medication treatments, heart function classification, etc.

（3） Admission Auxiliary Tests: Complete blood count, biochemical function tests, cardiac injury markers, NT-proBNP, glycated hemoglobin, ECG, echocardiogram, lower limb vascular ultrasound, etc.

（4） Daily Activity Assessment: Smoking, alcohol consumption, Barthel Index (BI), SPBB score, PHQ-9, GAD-7, Perme score, ADL score, SF-36 score, etc.

**Baseline Indicators and Observation Items**

（1） Demographic Data: Name, gender, age, date of birth, height, weight, etc.

（2） Biological History: Diagnosis, comorbidities, medication treatments, heart function classification, etc.

（3） Admission Auxiliary Tests: Complete blood count, biochemical function tests, cardiac injury markers, NT-proBNP, glycated hemoglobin, ECG, echocardiogram, lower limb vascular ultrasound, etc.

（4） Daily Activity Assessment: Smoking, alcohol consumption, Barthel Index (BI), SPBB score, PHQ-9, GAD-7, Perme score, ADL score, SF-36 score,etc.

（5） Following up: Patients will be followed regularly, with the time and cause of readmission and death recorded.

## 13. Standard Operating Procedures

Ultrasound examinations will be conducted by the Ultrasound Department of the Cardiovascular Hospital of Xiamen University. Blood samples will be collected and sent to the Laboratory Department for biochemical tests.

## 14. Statistical Methods

Data will be processed using SPSS 21.0. Quantitative data will be expressed as mean ± standard deviation, and categorical data as percentages (%). Group comparisons will be conducted using paired t-tests, and multiple sample means will be compared using ANOVA. Correlation analysis and multivariable linear regression will also be used, with a significance level set at $p < 0.05$.

## 15. Participant Management

1. All patients must sign a written informed consent form before participating in the study.
2. Patients will be enrolled based on the inclusion and exclusion criteria.
3. Patient privacy and safety will be protected throughout the study.

## 16. Specimen Management

Blood samples will be collected from participants for relevant indicator testing. Any remaining samples will be destroyed after use.

## 17. Drug and Equipment Management

Not applicable.

## 18. Data Management

All clinical data will be entered into specific CRF forms by Xuezhu Liu and Mengmeng Xue, who will also be responsible for data verification and analysis. The database will be managed by Linjing Wu.

## 19. Composition and Responsibilities of the Data Safety and Monitoring Committee

Data will be monitored by a third party.

## 20. Research Team

Principal Investigator: Linjing Wu

Study Supervisor: Cuilian Dai

Protocol Drafting and Data Analysis: Lijun Sun

Manuscript Writing: Jiahua Li

Data Collection: Wei Yan

Data Verification: Xuezhu Liu, Mengmeng Xue

## 21. Intellectual Property

All intellectual property generated from the research will belong to the Xiamen Cardiovascular Hospital, Xiamen University, with authorship determined by contributions to the research.

## 22. Publication Plan

Formulate an early activity plan for ADHF patients by early 2021; publish 1-2 academic papers.

## 23. Original Data Sharing Plan

Not applicable.

## 24. Post-Trial Treatment and Management for Participants

After the trial, if the participant's condition remains uncontrolled, the best treatment plan will be formulated based on their condition.

# Efficacy and Safety of Phase I Cardiac Rehabilitation in Patients with Acute Decompensated Heart Failure

## Statistical Analysis Plan

## V1.1

**Statistical Unit: Cardiovascular Research Institute, Xiamen Cardiovascular Hospital, Xiamen University**

**Sponsor: CCU, Xiamen Cardiovascular Hospital, Xiamen University**

**Publication Date: March 18, 2021**

# Contents

1. Introduction ........ 4
2. Clinical Study ........ 4
2.1 Objectives ........ 4
2.2 Content ........ 4
2.3 Primary outcomes ........ 5
2.4 Secondary outcomes ........ 5
2.5 Other outcomes ........ 5
3. Subjects ........ 5
3.1 Inclusion Criteria ........ 5
3.2 Exclusion Criteria ........ 6
4. General Statistical Considerations ........ 7
4.1 Sample Size Estimation ........ 7
4.2 Analysis Population ........ 7
4.3 Missing, Abnormal, and Outlier Data ........ 7
4.4 Statistical Analysis ........ 7
5. Statistical Analysis Indicators and Methods ........ 8
5.1 Demographic and Other Baseline Indicators ........ 8
5.2 Medications ........ 8
5.2 Outcomes ........ 8
6. Statistical Charts and Tables ........ 9
Figure 1. CONSORT Diagram ........ 9
Table 1 Subjects ........ 10
Table 2 Analysis of demographic data ........ 11
Table 3 Analysis of vital signs ........ 12
Table 4 Analysis of medical history ........ 14

Table 5 Analysis of lifestyle history ........ 15

Table 6 Rating and etiology of heart failure ........ 16

Table 7 Analysis of Echocardiographic data ........ 17

Table 8 Analysis of Laboratory findings ........ 18

Table 9 Analysis of lung functions ........ 20

Table 10 Analysis of Impedance cardiography findings........ 21

Table 11 Analysis of Nutritional assessments........ 23

Table 12 Primary Outcomes： SPBB ........ 24

Table 13 Primary Outcomes： Rehospitalization ........ 25

Table 14 Secondary Outcomes: life quantity and mobility ........ 26

# 1. Introduction

Acute decompensated heart failure (ADHF) refers to the acute onset or change of heart failure symptoms, which is the most common reason for hospitalization and cardiac death in heart failure patients. During hospitalization, ADHF patients experience decreased exercise tolerance due to prolonged bed rest, and often develop complications such as infections, pressure injuries, deep vein thrombosis, and acquired muscle weakness. Cardiac rehabilitation has become an important adjunct treatment for heart failure (HF) patients. Recent guidelines recommend early participation in safe and effective exercise training (or regular physical activity) to improve functional status (Ia level recommendation). However, research on early exercise rehabilitation for ADHF patients is still in its preliminary stages. There is no consensus on the timing, intensity, form, and duration of early exercise rehabilitation, and safety indicators are mainly based on complications, lacking tools to predict the safety of early activities. Therefore, it is necessary to explore scientific, systematic, safe, and effective exercise training programs.

This study aims to develop a Phase I cardiac rehabilitation program for intensive ADHF patients and evaluate its efficacy and safety by assessing physical fitness, mobility, quality of life, and heart and lung function.

# 2. Clinical Study

## 2.1 Objectives

This study aims to evaluate the safety and efficacy of early exercise rehabilitation in severe ADHF patients during the cardiac intensive care unit (CICU) phase.

## 2.2 Content

This study is a single-center, randomized, controlled, single-blind clinical study. It plans to recruit 120 severe ADHF patients in the CICU. The patients will be randomly divided into a control group and an intervention group. The intervention group will receive a customized cardiac rehabilitation program.

## 2.3 Primary outcomes

A. Short Physical Performance Battery (SPBB) score at discharge to evaluate the efficacy of early exercise rehabilitation.

B. Rehospitalization and mortality at 6 month to evaluate the safety of early exercise rehabilitation.

## 2.4 Secondary outcomes

A. Activities of daily living (ADL) at hospital discharge,

B. The Short-Form 36-Item Health Survey (SF-36) at 6-month post-discharge.

C. In-hospital mortality rate.

D. 6-month all-cause mortality rate.

## 2.5 Other outcomes

A．Cardiopulmonary function at hospital discharge

B．Other important clinical finding.

# 3. Subjects

## 3.1 Inclusion Criteria

（1）Age ≥ 18 years old；

（2）Acute decompensated heart failure was defined according to the “2021 ESC Guidelines for the diagnosis and treatment of acute and chronic heart failure”. Combined with symptoms, signs and laboratory results, and meet all the following conditions:

(2.1) At least one symptom was worsened from baseline: (a) dyspnea (dyspnea after exercising, paroxysmal nocturnal dyspnea or orthopnea), (b) venous congestion of systemic circulation (edema of lower extremities, liver congestion, ascites), and (c) tissue hypo-perfusion (oliguria or anuria, cold clammy limbs, consciousness disorder, hyper-lacticacidemia, or metabolic acidosis);

(2.2) At least one of the following signs of heart failure: (a) pulmonary edema on exam or by chest X ray, (b) Increased b-type natriuretic peptide or N-terminal prohormone BNP, and (c) abnormal cardiac structure and/or function by cardiac ultrasound;

（3）Patients who have New York Heart Association Cardiac Function class III or IV symptoms at admission.

（4）Clinical stability has been achieved for 24 h after optimizing treatment;

（5）Patient was independent with basic activities of daily living including the ability to ambulate independently (with or without the use of an assistive device) prior to admission.

## 3.2 Exclusion Criteria

(1) Acute coronary syndrome within 2 days; onset of chest pain within 8 h; or combined with mechanical complications;

(2) Fatal arrhythmia, high-grade atrioventricular block, new onset atrial fibrillation or atrial flutter;

(3) Severe aortic valve stenosis;

(4) Acute myocarditis, acute pericarditis, or acute infective endocarditis;

(5) Severe hypertrophic obstructive cardiomyopathy;

(6) Patients with chronic kidney disease stage 4 and 5 undergoing long-term hemodialysis or requiring long-term hemodialysis within the next 6 months;

(7) A recent embolism, atrium, or ventricular thrombosis, thrombophlebitis;

(8) Patients who require a pacemaker, implantable cardioverter defibrillator (ICD), or ventricular assist device, or those awaiting cardiac transplantation within the next 6 months;

(9) Those who had received standardized cardiac rehabilitation within the past 6 months;

(10) Severe cognitive impairment (MMSE $\leq$ 9 points);

(11) Inability or unwillingness to comply with the study requirements;

(12) Patients who dropped out or discharged during the study period.

# 4. General Statistical Considerations

## 4.1 Sample Size Estimation

According to the results of the previous study, the baseline SPPB sores was 6.0 ± 2.8 in CR group and 6.1 ± 2.6 in control group, the SPPB sores at 3 months was 8.3 ± 0.2 in CR group and 6.9 ± 0.2 in control group. To minimize both false positive and false negative errors, the type-I error rate (a) is set at 0.01 instead of 0.05 and the type-II error rate is set at 0.1 instead of 0.2. The sample size is calculated according to the following formula: n =2s2 × f (a,b)/(μ1-μ2)2 (19). The sample size of each group was estimated to be 44 per group. Taking approximate a 20% drop-off rate and a 7% missing rate into account, the final sample size was set to 60 per group. Therefore, a total of 120 subjects will be recruited in this study.

## 4.2 Analysis Population

Full Analysis Set (FAS): Defined by the intention-to-treat (ITT) principle, including all subjects who participated in the trial and actually used the study product.

All the statistical analysis was based on the FAS.

## 4.3 Missing, Abnormal, and Outlier Data

The total amount of missing data will be calculated. If the missing data are less than 5%, they will be imputed using statistical methods. If the missing data exceed 5%, a sensitivity analysis will be performed and reported in the statistical report. Data from patients who withdraw or drop out will still be included in the final statistical analysis, with detailed reasons for withdrawal or dropout reported in the statistical report.

## 4.4 Statistical Analysis

All statistical analyses will be conducted at a two-sided 0.05 significance level (unless otherwise specified). Statistical analysis will use SPSS and R software.

# 5. Statistical Analysis Indicators and Methods

## 5.1 Demographic and Other Baseline Indicators

Demographic data include gender, age (years), height (cm), weight (kg), BMI ($kg/m^2$). Other baseline indicators include vital signs, medical history, pre-admission echocardiography, laboratory tests (e.g., blood biochemistry, urinalysis, blood gas analysis), echocardiography, heart failure classification, medication history, and pulmonary function assessment. BMI is calculated as weight (kg) / $(\text{height (cm)} / 100)^2$. This section primarily involves descriptive statistics. Categorical data are described using frequency and percentage, while continuous data are described using mean, standard deviation, median, interquartile range (Q1, Q3), minimum, and maximum values.

## 5.2 Medications

A list of subjects using medications will be provided, including case number, age, gender, drug name, start date, end date, and whether the medication is ongoing.

## 5.2 Outcomes

（1）Continuous Variables

All continuous variables (e.g., SPPB score, ADL score, SF score) will be assessed using ANCOVA or the non-parametric test. Differences will be calculated, and 95% confidence intervals (CIs) will be presented.

Baseline measurements will be calculated as mean ± SD for normally distributed data, median with interquartile range (Q1-Q3) for non-normally distributed data.

（2）Categorical variables will be presented as counts and percentages.

（3）Time-to-event data will be analyzed using Cox proportional hazards regression and presented as Kaplan-Meier curves.

（4）Missing data were handled with Multiple Imputation by Chained Equations (MICE).

（5）Subgroup analysis and other outcome analyses will be considered exploratory. If important parameters are discovered, multiplicity will be accounted for in relation to the primary and secondary outcomes.

# 6. Statistical Charts and Tables

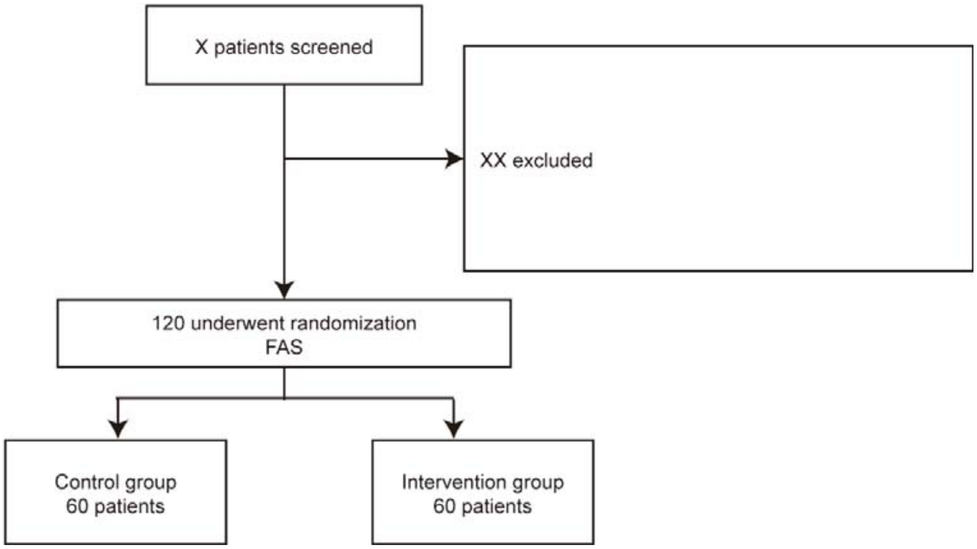


**Figure 1. CONSORT Diagram**

**Table 1 Subjects**

| Parameter | n，% |
|---|---|
| Inclusion | |
| Exclusion | |
| (1) Acute coronary syndrome within 2 days; onset of chest pain within 8 h; or combined with mechanical complications; | |
| (2) Fatal arrhythmia, high-grade atrioventricular block, new onset atrial fibrillation or atrial flutter; | |
| (3) Severe aortic valve stenosis; | |
| (4) Acute myocarditis, acute pericarditis, or acute infective endocarditis; | |
| (5) Severe hypertrophic obstructive cardiomyopathy; | |
| (6) Patients with chronic kidney disease stage 4 and 5 undergoing long-term hemodialysis or requiring long-term hemodialysis within the next 6 months; | |
| (7) A recent embolism, atrium, or ventricular thrombosis, thrombophlebitis; | |
| (8) Patients who require a pacemaker, implantable cardioverter defibrillator (ICD), or ventricular assist device, or those awaiting cardiac transplantation within the next 6 months; | |
| (9) Those who had received standardized cardiac rehabilitation within the past 6 months; | |
| (10) Severe cognitive impairment (MMSE ≤ 9 points); | |
| (11) Inability or unwillingness to comply with the study requirements; | |
| (12) Patients who dropped out or discharged during the study period. | |

**Table 2 Analysis of demographic data**

| Parameter | Value | Statistical results |
|---|---|---|
| Age (years) | Number of cases | |
| | Mean ± standard deviation | |
| | Median | |
| | Q1;Q3 | |
| | Minimum; Maximum | |
| Sex | Number of cases | |
| | Mean ± standard deviation | |
| | Median | |
| | Q1;Q3 | |
| | Minimum; Maximum | |
| Height (cm) | Number of cases | |
| | Mean ± standard deviation | |
| | Median | |
| | Q1;Q3 | |
| | Minimum; Maximum | |
| Body weight (kg) | Number of cases | |
| | Mean ± standard deviation | |
| | Median | |
| | Q1;Q3 | |
| | Minimum; Maximum | |
| Body mass index ($kg/m^2$) | Number of cases | |
| | Mean ± standard deviation | |
| | Median | |
| | Q1;Q3 | |
| | Minimum; Maximum | |

**Table 3 Analysis of vital signs**

| Parameter | Value | Statistical results |
|---|---|---|
| Systolic blood pressure (mmHg) | Number of cases | |
| | Mean ± standard deviation | |
| | Median | |
| | Q1;Q3 | |
| | Minimum; Maximum | |
| Diastolic blood pressure (mmHg) | Number of cases | |
| | Mean ± standard deviation | |
| | Median | |
| | Q1;Q3 | |
| | Minimum; Maximum | |
| Heart rate (beats per min) | Number of cases | |
| | Mean ± standard deviation | |
| | Median | |
| | Q1;Q3 | |
| | Minimum; Maximum | |
| Blood pH, n (%) | Number of cases | |
| | Mean ± standard deviation | |
| | Median | |
| | Q1;Q3 | |
| | Minimum; Maximum | |
| $PaO_2/FiO_2$ | Number of cases | |
| | Mean ± standard deviation | |
| | Median | |
| | Q1;Q3 | |
| | Minimum; Maximum | |
| Pulmonary crackles | Number of cases | |
| | Mean ± standard deviation | |

| | |
|---|---|
| | Median |
| | Q1;Q3 |
| | Minimum; Maximum |

**Table 4 Analysis of medical history**

| Parameter | Value | Statistical results |
|---|---|---|
| Diabetes mellitus | Number of cases | |
| | Present | |
| | Absent | |
| History of cerebrovascular disease | Number of cases | |
| | Present | |
| | Absent | |
| Chronic obstructive pulmonary disease | Number of cases | |
| | Present | |
| | Absent | |
| Chronic kidney disease | Number of cases | |
| | Present | |
| | Absent | |
| Prior hospitalization due to heart failure | Number of cases | |
| | Present | |
| | Absent | |

**Table 5 Analysis of lifestyle history**

| Parameter | Value | Statistical results |
|---|---|---|
| Smoking history, | Number of cases | |
| | Never | |
| | Former | |
| | Current | |
| Alcohol history | Number of cases | |
| | Never | |
| | Former | |
| | Current | |

**Table 6 Rating and etiology of heart failure**

| Parameter | Value | Statistical results |
|---|---|---|
| New York heart association functional class | Number of cases | |
| | I | |
| | II | |
| | III | |
| | IV | |
| Etiology | Number of cases | |
| | Ischemic heart disease | |
| | Valvular disease | |
| | Hypertension | |
| | Atrial fibrillation | |
| | Cardiomyopathy | |
| | Others | |

**Table 7 Analysis of Echocardiographic data**

| Parameter | Value | Statistical results |
|---|---|---|
| Left ventricle ejection fraction (%) | **Baseline & endpoints** | |
| | Mean ± standard deviation | |
| | Median | |
| | Q1;Q3 | |
| | Minimum; Maximum | |
| Left ventricle diameter (mm) | **Baseline & endpoints** | |
| | Mean ± standard deviation | |
| | Median | |
| | Q1;Q3 | |
| | Minimum; Maximum | |

**Table 8 Analysis of Laboratory findings**

| Parameter | Value | Statistical results |
|---|---|---|
| NT-proBNP, pg/ml | **Baseline & endpoints** | |
| | Mean ± standard deviation | |
| | Median | |
| | Q1;Q3 | |
| | Minimum; Maximum | |
| hs-cTnT, µg/ml | **Baseline & endpoints** | |
| | Mean ± standard deviation | |
| | Median | |
| | Q1;Q3 | |
| | Minimum; Maximum | |
| Triglyceride, mmol/L | **Baseline & endpoints** | |
| | Mean ± standard deviation | |
| | Median | |
| | Q1;Q3 | |
| | Minimum; Maximum | |
| Cholesterol, mmol/L | **Baseline & endpoints** | |
| | Mean ± standard deviation | |
| | Median | |
| | Q1;Q3 | |
| | Minimum; Maximum | |
| Low-density lipoprotein cholesterol, mmol/L | **Baseline & endpoints** | |
| | Mean ± standard deviation | |
| | Median | |
| | Q1;Q3 | |
| | Minimum; Maximum | |

| | |
|---|---|
| Serum creatinine, μg/ml | **Baseline & endpoints** |
| | Mean ± standard deviation |
| | Median |
| | Q1;Q3 |
| | Minimum; Maximum |
| Estimated glomerular filtration rate, ml/min/1.73 m² | **Baseline & endpoints** |
| | Mean ± standard deviation |
| | Median |
| | Q1;Q3 |
| | Minimum; Maximum |

**Table 9 Analysis of lung functions**

| Parameter | Value | Statistical results |
|---|---|---|
| Maximal voluntary ventilation, L/min | **Baseline & endpoints** | |
| | Mean ± standard deviation | |
| | Median | |
| | Q1;Q3 | |
| | Minimum; Maximum | |
| Forced Expiratory Volume in one second to Forced Vital Capacity ratio | **Baseline & endpoints** | |
| | Mean ± standard deviation | |
| | Median | |
| | Q1;Q3 | |
| | Minimum; Maximum | |
| Forceful expiratory spirometry, L | **Baseline & endpoints** | |
| | Mean ± standard deviation | |
| | Median | |
| | Q1;Q3 | |
| | Minimum; Maximum | |

**Table 10 Analysis of Impedance cardiography findings**

| Parameter | Value | Statistical results |
|---|---|---|
| Left ventricular ejection time, ms | **Baseline & endpoints** | |
| | Mean ± standard deviation | |
| | Median | |
| | Q1;Q3 | |
| | Minimum; Maximum | |
| Stroke volume, ml | **Baseline & endpoints** | |
| | Mean ± standard deviation | |
| | Median | |
| | Q1;Q3 | |
| | Minimum; Maximum | |
| Cardiac output, L/min | **Baseline & endpoints** | |
| | Mean ± standard deviation | |
| | Median | |
| | Q1;Q3 | |
| | Minimum; Maximum | |
| Cardiac index,, L/min/$m^2$ | **Baseline & endpoints** | |
| | Mean ± standard deviation | |
| | Median | |
| | Q1;Q3 | |
| | Minimum; Maximum | |
| Cardiac contractility index | **Baseline & endpoints** | |
| | Mean ± standard deviation | |
| | Median | |
| | Q1;Q3 | |
| | Minimum; Maximum | |
| Early diastolic filling rate, % | **Baseline & endpoints** | |
| | Mean ± standard deviation | |

| | |
|---|---|
| | Median |
| | Q1;Q3 |
| | Minimum; Maximum |
| Left heart work index, | **Baseline & endpoints** |
| $kg \cdot m/m^2$ | Mean ± standard deviation |
| | Median |
| | Q1;Q3 |
| | Minimum; Maximum |
| Systemic vascular resistance | **Baseline & endpoints** |
| index, $dyn \cdot s/cm^5 \cdot m^2$ | Mean ± standard deviation |
| | Median |
| | Q1;Q3 |
| | Minimum; Maximum |
| Ventricular systolic waves | **Baseline & endpoints** |
| | Mean ± standard deviation |
| | Median |
| | Q1;Q3 |
| | Minimum; Maximum |
| Ventricular diastolic waves | **Baseline & endpoints** |
| | Mean ± standard deviation |
| | Median |
| | Q1;Q3 |
| | Minimum; Maximum |

**Table 11 Analysis of Nutritional assessments**

| Parameter | Value | Statistical results |
|---|---|---|
| Nutrition Risk Screening 2002 | Number of cases | |
| | <3 | |
| | >=3 | |
| Mini Nutritional Assessment Short-Form | Number of cases | |
| | Normal nutritional status | |
| | At risk | |
| | Malnourished | |

**Table 12 Primary Outcomes：SPBB**

| Parameter | Value | Statistical results |
|---|---|---|
| SPBB | **Baseline & endpoints** | ANCOVA or the |
| | Mean ± standard deviation | non-parametric test |
| | Median | |
| | Q1;Q3 | |
| | Minimum; Maximum | |
| Balance | **Baseline & endpoints** | / |
| | Mean ± standard deviation | |
| | Median | |
| | Q1;Q3 | |
| | Minimum; Maximum | |
| Gait speed | **Baseline & endpoints** | / |
| | Mean ± standard deviation | |
| | Median | |
| | Q1;Q3 | |
| | Minimum; Maximum | |
| Chair rise | **Baseline & endpoints** | / |
| | Mean ± standard deviation | |
| | Median | |
| | Q1;Q3 | |
| | Minimum; Maximum | |

**Table 13 Primary Outcomes： Rehospitalization**

| Parameter | Value | Statistical results |
|---|---|---|
| Rehospitalization for any cause | Counts and percentages | Cox proportional hazards regression<br>K-M curve |

**Table 14 Secondary Outcomes: life quantity and mobility**

| Parameter | Value | Statistical results |
|---|---|---|
| ADL | **Baseline & endpoints**<br>Mean ± standard deviation<br>Median<br>Q1;Q3<br>Minimum; Maximum | ANCOVA or the non-parametric test |
| SF-36 | **Baseline & endpoints**<br>Mean ± standard deviation<br>Median<br>Q1;Q3<br>Minimum; Maximum | ANCOVA or the non-parametric test |
| Death | Counts and percentages | Cox proportional hazards regression<br>K-M curve |
| In-hospital mortality rate | Counts and percentages | Cox proportional hazards regression<br>K-M curve |

# 厦门大学附属心血管病医院医学伦理委员会
# 伦理审查同意函

| 批件号 | （2021）医伦科第（6）号 | | |
|---|---|---|---|
| 项目名称 | 急性失代偿性心力衰竭患者 I 期心脏康复的有效性和安全性研究 | | |
| 申办单位 | 厦门大学附属心血管病医院 | | |
| 本院主要研究者 | 吴林静 | 科室 | 冠心病监护室（CCU） |
| 审查类别及方式 | 初始审查 ■会议审查（2021 年 03 月 01 日，作必要修正后同意）4 楼行政会议室<br>复审 ■快速审查（2021 年 03 月 23 日） | | |
| 审查文件 | 详见附录 | | |
| 审查意见 | 同意按照审查的文件进行本项研究。 | | |

根据《涉及人的生物医学研究伦理审查办法》（2016）、《药物临床试验质量管理规范（2020）》、《药物临床试验伦理审查工作指导原则（2010）》、《医疗器械临床试验质量管理规范（2016）》、《医疗器械临床试验规定（2004）》、《体外诊断试剂临床研究技术指导原则（2007）》、《关于印发医疗卫生机构开展临床研究项目管理办法的通知(2014)》、《涉及人的临床研究伦理审查委员会建设指南（2020 版）》、《赫尔辛基宣言》和《人体生物医学研究国际道德指南》的伦理原则，经本伦理委员会审查，同意按所审查的文件开展本项研究。

请遵守 GCP 原则，遵循伦理委员会审查同意的方案开展临床研究，保护受试者的健康与权利。属《人类遗传资源采集、收集、买卖、出口、 出境审批行政许可事项》规定范畴的研究，获得伦理审查同意后应按相关法规进行审批，获得中国人类遗传资源管理工作办公室批准后及时将批准文件交伦理委员会备案后方可实施。属《需进行临床试验审批的第三类医疗器械目录》内医疗器械的临床试验，获得伦理审查同意后应按相关法规进行审批，获得国家食药监局批准后及时将批准文件交伦理委员会备案后方可实施。

研究过程中若变更主要研究者，或对临床方案、知情同意书、招募材料等的任何修改，请申请人提交修正案审查申请。

发生严重不良事件，请申请人及时提交不良事件报告。

请按照伦理委员会规定的年度/定期跟踪审查频率，申请人在截止日期前 1 个月提交研究进展报告；申办者应当向组织单位伦理委员会提供各中心研究进展的汇总报告；当出现任何可能显著影响试验进行或增加受试者危险的情况时，请申请人及时向伦理委员会提交书面报告。

研究纳入了不符合纳入标准或符合排除标准的受试者，符合终止试验规定而未让受试者退出的研究，给予错误治疗或剂量，给予方案禁止的合并用药等没有遵从方案开展研究的情况；或者可能对受试者的权益/健康以及研究的科学性造成不良影响等违背 GCP 原则的情况，请申办者/监察员/研究者提交违背方案报告。

申请人暂停或提前终止临床研究，请及时提交暂停或终止研究报告。

完成临床研究，请申请人提交结题报告。

| 年度/定期跟踪审查频率 | 12 个月 | | |
|---|---|---|---|
| 有效期 | 该批件有效期 1 年（自审查同意之日起），如试验 1 年未实施需要提出延长有效期申请。 | | |
| 联系人 | 王仁霁 | 联系电话 | 0592-2292562 |
| 伦理委员会(盖章) | 厦门大学附属心血管病医院医学伦理委员会 | | |
| 主任委员签字<br>（被授权委员） | [illegible] | | |
| 日期 | 2021.3.23 | | |

# 厦门大学附属心血管病医院医学伦理委员会附录

| 批件号 | （2021）医伦科第（6）号 |
|---|---|
| 项目名称 | 急性失代偿性心力衰竭患者Ⅰ期心脏康复的有效性和安全性研究 |
| 申办单位 | 厦门大学附属心血管病医院 |

本次审查的文件清单：

1.复审申请表
2.临床研究方案（版本号：1.1，版本日期：2021-03-18）
3.知情同意书（版本号：1.1，版本日期：2021-03-18）
4.主要研究者简历
5.病例报告表（版本号：V1.0，版本日期：2021-01-01）

本伦理委员会的人员组成和工作程序符合中国 GCP 以及国家相关规定。

# Ethics Committee of Xiamen Cardiovascular Hospital, Xiamen University

## Ethical review consent letter

| | | | |
|---|---|---|---|
| Approval No. | (2021) YLK. (6) | | |
| Project name | Efficacy and Safety of Phase I Cardiac Rehabilitation in Patients with Acute Decompensated Heart Failure | | |
| Applicant | Xiamen Cardiovascular Hospital, Xiamen University | | |
| PI | Linjing Wu | Department | Cardiac intensive care unit |
| Types and methods of review | Initial Review: Meeting Review (March 01, 2021, approved after necessary amendments) 4th Floor Administrative Meeting Room<br>Re-examination: Quick Review (March 23, 2021 | | |
| Review documents | In Appendix | | |
| Review Comments | Agree to conduct this study according to the reviewed documents. | | |

In accordance with the ethical principles outlined in the "Ethical Review Measures for Biomedical Research Involving Humans" (2016), "Good Clinical Practice for Drug Clinical Trials" (2020), "Guidelines for Ethical Review of Drug Clinical Trials" (2010), "Good Clinical Practice for Medical Device Clinical Trials" (2016), "Medical Device Clinical Trials Regulations" (2004), "Technical Guidelines for Clinical Research of In Vitro Diagnostic Reagents" (2007), "Notice on Issuing the Management Measures for Clinical Research Projects Conducted by Medical and Health Institutions" (2014), "Guidelines for the Establishment of Ethical Review Committees for Clinical Research Involving Humans" (2020 Edition), "Declaration of Helsinki," and "International Ethical Guidelines for Biomedical Research Involving Humans," this research has been reviewed and approved.

Please adhere to Good Clinical Practice (GCP) principles and conduct clinical research according to the plan approved by the ethics committee, ensuring the protection of subjects' health and rights. Research involving human genetic resources, as outlined in the "Administrative Licensing Matters for the Collection, Sale, Export, and Exit of Human Genetic Resources," must be approved by the China Human Genetic Resources Management Office following an ethical review. Implementation is contingent upon receiving this approval and the timely submission of documentation to the ethics committee for filing. Additionally, clinical trials of medical devices listed in the "Catalogue of Class III Medical Devices Requiring Clinical Trial Approval" must be approved by the State Food and Drug Administration after obtaining ethical review approval. These trials can only proceed after securing this approval and submitting the necessary documents to the ethics committee for filing.

If there is a change in the principal investigator during the study, or if there are any modifications to the clinical protocol, informed consent form, recruitment materials, or other study documents, the applicant must submit an application for an amendment review.

If a serious adverse event occurs, please submit an adverse event report promptly.

Please adhere to the annual or periodic follow-up review frequency stipulated by the ethics committee. Submit a research progress report one month before the deadline. The sponsor must provide a summary report on the research progress from each center to the ethics committee. If any issues arise that could significantly impact the trial or increase risk to subjects, a written

<table>
<tr><td colspan="4">report must be submitted to the ethics committee promptly.<br>In cases where the study includes subjects who do not meet the inclusion criteria, fail to meet the exclusion criteria, or where protocol violations occur (e.g., incorrect treatment or dosage, administration of prohibited medications), and if these issues could adversely affect the subjects' rights, health, or the scientific validity of the research, the sponsor, ombudsman, or researcher must submit a protocol violation report.<br>If the applicant suspends or terminates the clinical research early, a suspension or termination report must be submitted promptly, along with a completion report once the research is concluded.</td></tr>
<tr><td>Annual/periodic follow-up review frequency<br><br>Validity</td><td colspan="3">12 months<br><br>The approval is valid for one year (starting from the date of review and approval). If the test is not carried out within one year, an application for extension of the validity period must be submitted.</td></tr>
<tr><td>Contact</td><td>Renji Wang</td><td>contact number</td><td>0592 - 2292562</td></tr>
<tr><td>Ethics Committee (seal)</td><td colspan="3">Ethics Committee of Xiamen Cardiovascular Hospital, Xiamen University</td></tr>
<tr><td>Signature of Chairman<br>( Authorized Member)</td><td colspan="3"></td></tr>
<tr><td>Date</td><td colspan="3"></td></tr>
</table>

# Appendix

| | |
|---|---|
| Approval No. | (2021) Medical Science No. (6) |
| project name | A study on the effectiveness and safety of phase I cardiac rehabilitation in patients with acute decompensated heart failure |
| Applicant | Xiamen University Cardiovascular Hospital |
| List of documents for this review:<br>1. Review Application Form<br>2. Clinical study protocol (version number: 1.1 , version date : 2021-03-18 )<br>3. Informed Consent Form (version number: 1.1, version date : 2021-03-18 )<br>4. Resume of the principal investigator<br>5. Case Report Form ( Version Number : V 1.0 , Version Date : 2021-01-01 ) | |
| The personnel composition and working procedures of this ethics committee comply with China's GCP and relevant national regulations. | |

# ChiCTR2100050151 版本V1.3 版本创建时间2022/04/24 06:01:10 中国临床试验注册中心

| | |
|---|---|
| 审核状态:<br>Project audit state: | 通过审核<br>Successful |
| 注册号:<br>Registration number: | ChiCTR2100050151 |
| 最近更新日期:<br>Date of Last Refreshed on: | 2022-03-19 |
| 注册时间:<br>Date of Registration: | 2021-08-19 |
| 注册号状态: | 预注册 |
| Registration Status: | Prospective registration |
| 注册题目: | 急性失代偿性心力衰竭患者I期心脏康复的有效性和安全性研究 |
| Public title: | The efficacy and safety of stage I cardiac rehabilitation in patients with acute decompensated heart failure |
| 注册题目简写: | |
| English Acronym: | |
| 研究课题的正式科学名称: | 急性失代偿性心力衰竭患者I期心脏康复的有效性和安全性研究 |
| Scientific title: | The efficacy and safety of stage I cardiac rehabilitation in patients with acute decompensated heart failure |
| 研究课题代号(代码):<br>Study subject ID: | |
| 在二级注册机构或其它机构的注册号:<br>The registration number of the Partner Registry or other register: | |

| | | | |
|---|---|---|---|
| 申请注册联系人: | 张鹏 | 研究负责人: | 吴林静 |
| Applicant: | Zhang Peng | Study leader: | Wu Linjing |
| 申请注册联系人电话:<br>Applicant telephone: | +86 592 2293272 | 研究负责人电话:<br>Study leader's telephone: | +86 592 2293057 |
| 申请注册联系人传真 :<br>Applicant Fax: | | 研究负责人传真:<br>Study leader's fax: | |
| 申请注册联系人电子邮件:<br>Applicant E-mail: | xiaxincr@163.com | 研究负责人电子邮件:<br>Study leader's E-mail: | 3620883@qq.com |
| 申请单位网址(自愿提供):<br>Applicant website(voluntary supply): | | 研究负责人网址(自愿提供):<br>Study leader's website(voluntary supply): | |
| 申请注册联系人通讯地址: | 福建省厦门市湖里区金山路2999号 | 研究负责人通讯地址: | 福建省厦门市湖里区金山路2999号 |
| Applicant address: | 2999 Jinshan Road, Huli District, Xiamen, Fujian, China | Study leader's address: | 2999 Jinshan Road, Huli District, Xiamen, Fujian, China |
| 申请注册联系人邮政编码:<br>Applicant postcode: | | 研究负责人邮政编码:<br>Study leader's postcode: | |
| 申请人所在单位: | 厦门大学附属心血管病医院 | | |
| Applicant's institution: | Xiamen Cardiovascular Hospital of Xiamen University | | |
| 研究负责人所在单位: | 厦门大学附属心血管病医院 | | |
| Affiliation of the Leader: | Xiamen Cardiovascular Hospital of Xiamen University | | |

| | | | |
|---|---|---|---|
| 是否获伦理委员会批准: | 是 | | |
| Approved by ethic committee: | Yes | | |
| 伦理委员会批件文号:<br>Approved No. of ethic committee: | （2021）医伦科第（6）号 | 伦理委员会批件附件:<br>Approved file of Ethical Committee: | 查看附件View |
| 批准本研究的伦理委员会名称: | 厦门大学附属心血管病医院医学伦理委员会 | | |
| Name of the ethic committee: | Ethics Committee of Xiamen Cardiovascular Hospital of Xiamen University | | |
| 伦理委员会批准日期:<br>Date of approved by ethic committee: | 2021-03-25 | | |
| 伦理委员会联系人: | 王仁霁 | | |
| Contact Name of the ethic committee: | Wang Renji | | |

| | |
|---|---|
| 伦理委员会联系地址： | 福建省厦门市湖里区金山路2999号 |
| Contact Address of the ethic committee: | 2999 Jinshan Road, Huli District, Xiamen, Fujian, China |
| 伦理委员会联系人电话：<br>Contact phone of the ethic committee: | 伦理委员会联系人邮箱：<br>Contact email of the ethic committee: |

| | |
|---|---|
| 研究实施负责（组长）单位： | 厦门大学附属心血管病医院 |
| Primary sponsor: | Xiamen Cardiovascular Hospital of Xiamen University |
| 研究实施负责（组长）单位地址： | 福建省厦门市湖里区金山路2999号 |
| Primary sponsor's address: | 2999 Jinshan Road, Huli District, Xiamen, Fujian, China |

试验主办单位(项目批准或申办者)：
Secondary sponsor:

| | | | | | |
|---|---|---|---|---|---|
| 国家： | 中国 | 省(直辖市)： | 福建 | 市(区县)： | 厦门 |
| Country: | China | Province: | Fujian | City: | Xiamen |
| 单位(医院)： | 厦门大学附属心血管病医院 | 具体地址： | 湖里区金山路2999号 | | |
| Institution hospital: | Xiamen Cardiovascular Hospital of Xiamen University | Address: | 2999 Jinshan Road, Huli District | | |

| | |
|---|---|
| 经费或物资来源： | 厦门市医疗卫生指导项目 |
| Source(s) of funding: | Medical and Health Guidance Program of Xiamen |
| 研究疾病： | 心力衰竭 |
| Target disease: | heart failure |
| 研究疾病代码： | |
| Target disease code: | |
| 研究类型： | 干预性研究 |
| Study type: | Interventional study |
| 研究所处阶段： | I期临床试验 |
| Study phase: | 1 |
| 研究设计： | 随机平行对照 |
| Study design: | Parallel |
| 研究目的： | 1.探索急性失代偿性心力衰竭患者I期心脏康复的安全性和有效性； 2.明确I期心脏康复对急性失代偿性心力衰竭患者再住院率、生活自理能力、心肺功能以及生活质量的影响。 |
| Objectives of Study: | 1. Explore the safety and efficacy of phase I cardiac rehabilitation in patients with acute decompensated heart failure; 2. Clarify the effect of phase I cardiac rehabilitation on the rate of rehospitalization, self-care ability, cardiopulmonary function, and quality of life in patients with acute decompensated heart failure. |
| 药物成份或治疗方案详述： | |
| Description for medicine or protocol of treatment in detail: | |
| 纳入标准： | 1. 年龄≥18岁；<br>2. 根据《2021欧洲心脏病学会心力衰竭协会共识：心力衰竭调整药物治疗的患者特征》的ADHF的定义，结合症状、体征和检验检查，并满足以下所有条件；<br>(1)至少以下一项心衰症状或体征恶化：<br>1)呼吸困难（劳力性呼吸困难，夜间阵发性呼吸困难,端坐呼吸）；<br>2)体循环淤血表现（下肢水肿、肝淤血、腹腔积液）；<br>3)组织低灌注的表现 (少尿或无尿、四肢湿冷、意识障碍、高乳酸血症、代谢性酸中毒)；<br>(2) 至少以下检查有一项符合心衰表现：<br>1)体格检查或胸部X片有急性肺水肿的表现；<br>2)BNP或NT-proBNP升高；<br>3)心脏彩超提示心脏结构和/或功能异常；<br>3. 经研究者判断症状和体征符合ADHF表现；<br>4. 入院时符合纽约心脏协会心功能III或IV级标准；<br>5. 经过心衰药物优化治疗后，临床症状稳定24小时；<br>6. 入院前能够基本完成日常生活活动及具备行走的能力（有或没有使用辅助设备）。 |
| Inclusion criteria | 1. Age >= 18 years old;<br>2. Acute decompensated heart failure was defined according to the patient profiling in heart failure for tailoring medical therapy, combined with symptoms, signs and laboratory results, and meet all the following conditions;<br>(1) At least one symptom was worsened from baseline:<br>1) Dyspnea (dyspnea after exercising, paroxysmal nocturnal dyspnea or orthopnea);<br>2) Venous congestion of systemic circulation (edema of lower extremities, liver congestion, ascites);<br>3) Tissue hypoperfusion (oliguria or anuria, cold clammy limbs, consciousness disorder, hyperlacticacidemia or metabolic acidosis);<br>(2) At least one of the following signs of heart failure:<br>1) Pulmonary edema on exam or by chest X ray;<br>2) Increased b-type natriuretic peptide or N-terminal prohormone BNP;<br>3) Abnormal cardiac structure and/or function by cardiac ultrasound;<br>3. The symptoms and signs are consistent with acute decompensated heart failure judged by the investigator;<br>4. Patients who have New York Heart Association Cardiac Function class III or IV symptoms at admission;<br>5. Clinical stability has been achieved for 24 hours after optimizing treatment; |

| | |
|---|---|
| | 6. Patient was independent with basic activities of daily living including the ability to ambulate independently (with or without the use of an assistive device) prior to admission. |
| 排除标准: | 1. 急性冠状动脉综合征2天内，过去8小时内新发或再发胸痛，合并机械并发症;<br>2. 致命性心律失常、高度房室传导阻滞、新发心房颤动或心房扑动;<br>3. 有症状的主动脉瓣重度狭窄;<br>4. 急性心肌炎，急性心包炎或急性感染性心内膜炎;<br>5. 严重的肥厚型梗阻性心肌病;<br>6. 慢性肾功能不全4期和5期，并长期进行血液透析或预计在6个月内需长期血液透析;<br>7. 近期栓塞、心房或心室内血栓、血栓性静脉炎;<br>8. 预计在6个月内植入起搏器、植入式心律转复除颤器、心室辅助装置或心脏移植手术;<br>9. 既往6个月内参加规范化的心脏康复;<br>10. 重度认知障碍（MMSE≤9分）;<br>11. 无法或不愿意遵守研究要求;<br>12. 研究期间中途退出或出院者。 |
| Exclusion criteria: | 1. Acute coronary syndrome within 2 days, onset of chest pain within 8 hours; or combined with mechanical complications;<br>2. Fatal arrhythmia, high-grade atrioventricular block, new onset atrial fibrillation or atrial flutter;<br>3. Severe aortic valve stenosis;<br>4. Acute myocarditis, acute pericarditis, or acute infective endocarditis;<br>5. Severe hypertrophic obstructive cardiomyopathy;<br>6. Patients with chronic kidney disease stage 4 and 5 undergoing long-term hemodialysis or requiring long-term hemodialysis within the next 6 months;<br>7. A recent embolism, atrium or ventricular thrombosis, thrombophlebitis;<br>8. Patients who require a pacemaker, implantable cardioverter defibrillator (ICD), or ventricular assist device, or those awaiting cardiac transplantation within the next 6 months;<br>9. Those who had received standardized cardiac rehabilitation within the past 6 months;<br>10. Severe cognitive impairment (MMSE<=9 points);<br>11. Inability or unwillingness to comply with the study requirements;<br>12. Patients who dropped out or discharged during the study period. |

| | | | |
|---|---|---|---|
| 研究实施时间:<br>Study execute time: | 从 From 2021-09-01至 To 2023-09-01 | 征募观察对象时间:<br>Recruiting time: | 从 From 2021-09-01 至 To 2023-09-01 |

干预措施:
Interventions:

| | | | |
|---|---|---|---|
| 组别: | 试验组 | 样本量: | 60 |
| Group: | experimental group | Sample size: | |
| 干预措施: | 心脏康复 | 干预措施代码: | |
| Intervention: | cardiac rehabilitation | Intervention code: | |
| 组别: | 对照组 | 样本量: | 60 |
| Group: | control group | Sample size: | |
| 干预措施: | 常规治疗 | 干预措施代码: | |
| Intervention: | common treatment | Intervention code: | |

研究实施地点:
Countries of recruitment and research settings:

| | | | | | |
|---|---|---|---|---|---|
| 国家: | 中国 | 省(直辖市): | 福建 | 市(区县): | 厦门 |
| Country: | China | Province: | Fujian | City: | Xiamen |
| 单位(医院): | 厦门大学附属心血管病医院 | 单位级别: | 三级 | | |
| Institution hospital: | Xiamen Cardiovascular Hospital of Xiamen University | Level of the institution: | Tertiary | | |

测量指标:
Outcomes:

| | | | |
|---|---|---|---|
| 指标中文名: | 简易体能状况量表 | 指标类型: | 主要指标 |
| Outcome: | short physical performance battery | Type: | Primary indicator |
| 测量时间点: | | 测量方法: | |
| Measure time point of outcome: | | Measure method: | |
| 指标中文名: | 心功能 | 指标类型: | 次要指标 |
| Outcome: | cardiac function | Type: | Secondary indicator |
| 测量时间点: | | 测量方法: | |
| Measure time point of outcome: | | Measure method: | |
| 指标中文名: | 心理评估 | 指标类型: | 次要指标 |
| Outcome: | psychological assessment | Type: | Secondary indicator |
| 测量时间点: | | 测量方法: | |
| Measure time point of outcome: | | Measure method: | |
| 指标中文名: | 生活质量 | 指标类型: | 次要指标 |

| | | | |
|---|---|---|---|
| Outcome： | the quality of life | Type： | Secondary indicator |
| 测量时间点： | | 测量方法： | |
| Measure time point of outcome： | | Measure method： | |

| 采集人体标本：<br>Collecting sample(s) from participants： | | | | |
|---|---|---|---|---|
| | 标本中文名： | 血液 | 组织： | |
| | Sample Name： | blood | Tissue： | |
| | 人体标本去向 | 使用后销毁 | 说明 | |
| | Fate of sample： | Destruction after use | Note： | |

| | | | |
|---|---|---|---|
| 征募研究对象情况：<br>Recruiting status： | 尚未开始<br>Not yet recruiting | 年龄范围：<br>Participant age： | 最小 Min age 18 岁 years<br>最大 Max age 岁 years |
| 性别： | 男女均可 | Gender： | Both |
| 随机方法（请说明由何人用什么方法产生随机序列）： | 随机序列表 | | |
| Randomization Procedure (please state who generates the random number sequence and by what method)： | Random sequence list | | |
| 是否公开试验完成后的统计结果：<br>Calculated Results after the Study Completed public access： | 公开/Public | | |
| 盲法： | | | |
| Blinding： | | | |
| 试验完成后的统计结果（上传文件）： | 点击下载 | | |
| Calculated Results after the Study Completed(upload file)： | download | | |
| 是否共享原始数据：<br>IPD sharing | 是Yes | | |
| 共享原始数据的方式（说明：请填入公开原始数据日期和方式，如采用网络平台，需填该网络平台名称和网址）： | 论文形式发表 | | |
| The way of sharing IPD”(include metadata and protocol, If use web-based public database, please provide the url)： | publish paper | | |
| 数据采集和管理（说明：数据采集和管理由两部分组成，一为病例记录表（Case Record Form, CRF），二为电子采集和管理系统(Electronic Data Capture, EDC)，如ResMan即为一种基于互联网的EDC： | 所有数据都会如实记录在CRF表格中,填写完毕的CRF数据将被输入本研究项目数据管理系统中。 | | |
| Data collection and Management (A standard data collection and management system include a CRF and an electronic data capture： | All data will be recorded in CRF and be inputted in data management system of this study | | |
| 数据与安全监察委员会：<br>Data and Safety Monitoring Committee： | 有/Yes | | |
