# Supplementary material for: Early Cardiac Rehabilitation for Critically Ill Patients With Acute Decompensated Heart Failure: A Randomized Clinical Trial
Source: JAMA Netw Open. 2025 Jul 30;8(7):e2524141. doi: 10.1001/jamanetworkopen.2025.24141 (PMC12311714; doi:10.1001/jamanetworkopen.2025.24141)
Supplement: Supplement 2. — eFigure 1. Length of Stay for Patients Before and After Randomization eFigure 2. Summary of the Study eTable 1. Assessment, Protocol, and Goals of the Tailored Cardiac Rehabilitation Program (AHF-CR) eTable 2. Key Characteristics of Patients at CICU Administration eTable 3. Characteristics of Physical Therapy During CICU Stay eTable 4. Supporting Outcomes About Cardiopulmonary Function at CICU Discharge eTable 5. Subgroup Analysis of the PERME Score [file jamanetwopen-e2524141-s002.pdf]

# Supplemental Online Content

Wu L, Li J, Zheng Y, et al. Early cardiac rehabilitation for critically ill patients with acute decompensated heart failure: a randomized clinical trial. *JAMA Netw Open*. 2025;8(7):e2524141. doi:10.1001/jamanetworkopen.2025.24141

**eFigure 1.** Length of Stay for Patients Before and After Randomization

**eFigure 2.** Summary of the Study

**eTable 1.** Assessment, Protocol, and Goals of the Tailored Cardiac Rehabilitation Program (AHF-CR)

**eTable 2.** Key Characteristics of Patients at CICU Administration

**eTable 3.** Characteristics of Physical Therapy During CICU Stay

**eTable 4.** Supporting Outcomes About Cardiopulmonary Function at CICU Discharge

**eTable 5.** Subgroup Analysis of the PERME Score

This supplemental material has been provided by the authors to give readers additional information about their work.

© 2025 Wu L et al. *JAMA Network Open*.

**eFigure 1.** Length of Stay for Patients Before and After Randomization

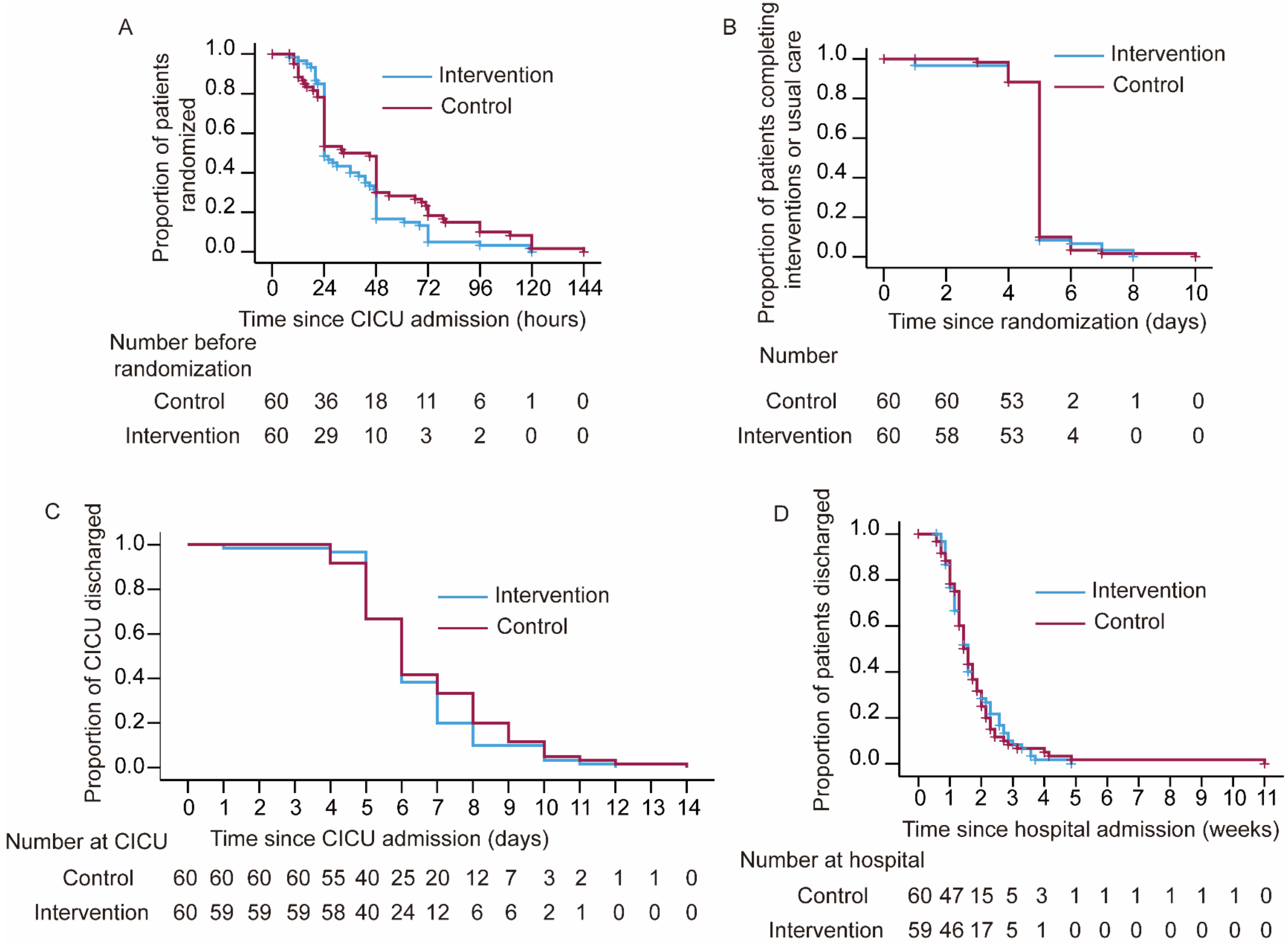


**(A)** Kaplan-Meier curve of patient stay in the CICU before randomization. **(B)** Kaplan-Meier curve of patients receiving either the intervention or usual care during their stay in the CICU. **(C)** Kaplan-Meier curve of patient stay in the CICU. **(D)** Kaplan-Meier curve of patient stay in the hospital. CICU = Cardiac Intensive Care Unit.

© 2025 Wu L et al. *JAMA Network Open*.

**eFigure 2.** Summary of the Study

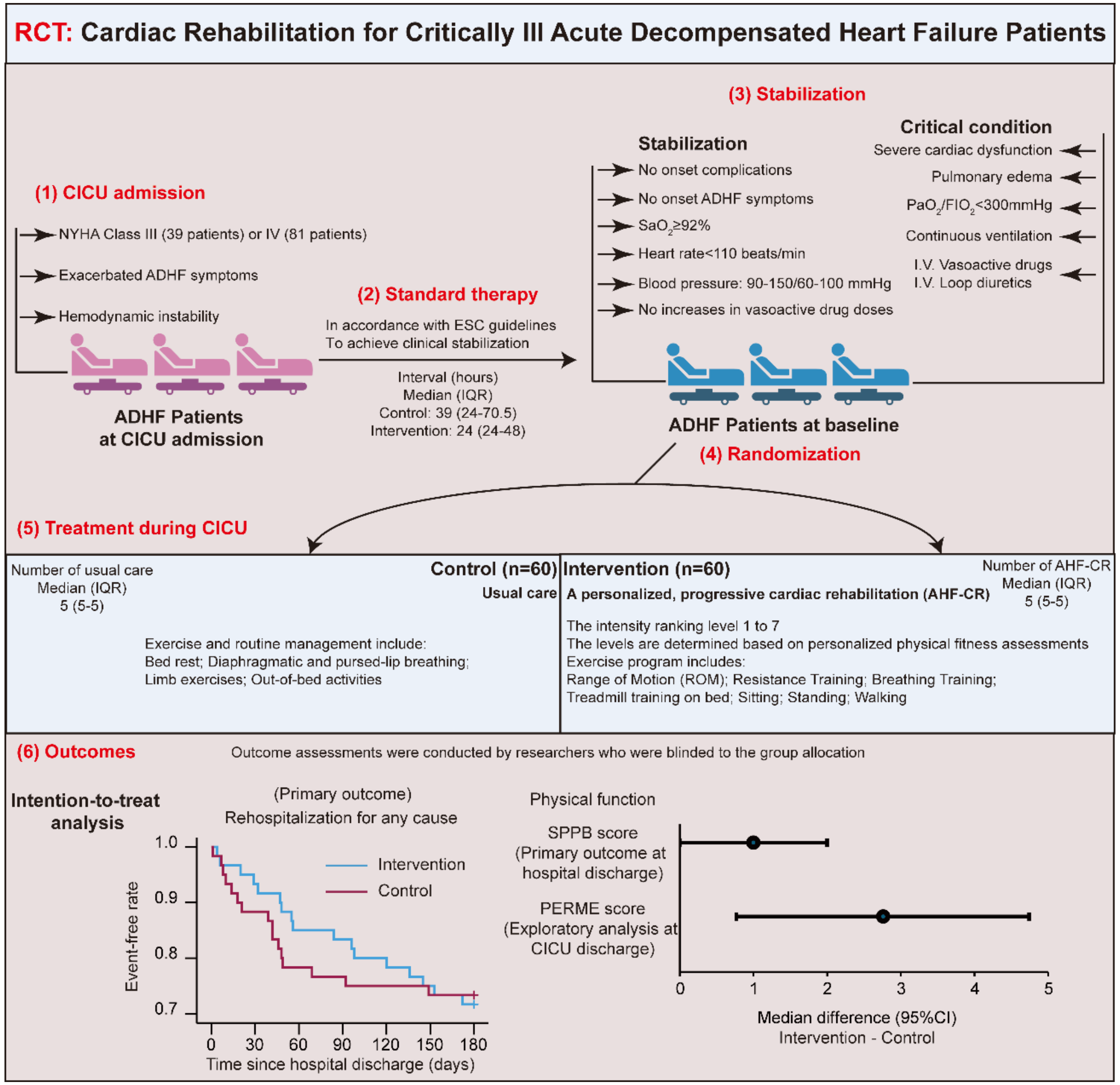


The patient characteristics and intervention details are presented. The Kaplan-Meier curve illustrates rehospitalization events within six months post-hospital discharge. Additionally, the median differences in SPPB and PERME scores between the intervention and control groups are reported. ADHF = acute decompensated heart failure; CICU = Cardiac Intensive Care Unit; NYHA = New York Heart Association; $PaO_2/FiO_2$ = ratio of arterial partial pressure of oxygen to fraction of inspired oxygen; PERME = Perme ICU Mobility; $SaO_2$ = blood oxygen saturation; SPPB = Short Physical Performance Battery.

© 2025 Wu L et al. *JAMA Network Open*.

**eTable 1.** Assessment, Protocol, and Goals of the Tailored Cardiac Rehabilitation Program (AHF-CR)[a]

| Fitness level | 1 | 2 | 3 | 4 | 5 | 6 | 7 |
|---|---|---|---|---|---|---|---|
| **Assessment** | | | | | | | |
| Mental states | Unconscious | Conscious | Conscious | Conscious | Conscious | Conscious | Conscious |
| Muscle strength [b] | Upper limb muscle strength < level 3 | Upper limb muscle strength ≥level 3 | Limb muscle strength ≥ level 3 | Limb muscle strength ≥ level 4 | Limb muscle strength ≥ level 4 | Lower limbs muscle strength = level 5 | Lower limbs muscle strength = level 5 |
| NYHA classification | III–IV | III–IV | III | II-III | II–III | II–III | II–III |
| **Protocol** | | | | | | | |
| ROM | Passive ROM once per day | Active/passive ROM once per day | Active ROM once per day | Active ROM once per day | Active ROM once per day | Active ROM once per day | Active ROM once per day |
| Treadmill training | Passive bed treadmill training for 10–20 min | Passive bed treadmill training for 10–20 min | Bed treadmill training for 10–20 min | Bed treadmill training for 10–20 min | Bed treadmill training for 10–20 min | Bed treadmill training for 10–20 min | Bed treadmill training for 10–20 min |
| Progressive breathing training | / | / | Yes | Yes | Yes | Yes | Yes |
| Progressive resistance training (15 repetitions per set, with a 2-minute rest between sets). | / | / | Elbow flexion and extension movements for 3 sets | Elbow flexion and extension movements for 3 sets and abdominal crunch for 1 set and hip bridge for 1 set | Elbow flexion and extension movements for 3 sets and abdominal crunch for 1 set and hip bridge for 1 set | Elbow flexion and extension movements for 3 sets and abdominal crunch for 1 set and hip bridge for 1 set | Elbow flexion and extension movements for 3 sets and abdominal crunch for 1 set and hip bridge for 1 set |
| Sitting in bed with the head of the bed elevated to more than 45° | / | 5 min, 2 times per day | 5 min, 2 times per day | 5 min, 2 times per day | 5 min, 2 times per day | 5 min, 2 times per day | 5 min, 2 times per day |
| Sitting at the edge of the bed | / | / | 5 min, 2 times per day | 5 min, 2 times per day | 5 min, 2 times per day | 5 min, 2 times per day | 5 min, 2 times per day |
| Standing and stepping | / | / | / | 2 min | 2 min | 5 min | 5 min |

© 2025 Wu L et al. *JAMA Network Open*.

| | | | | | | | |
|---|---|---|---|---|---|---|---|
| Bed to chair transfer | / | / | / | Sitting in a chair for 5 min per day, twice a day | Sitting in a chair for 5 min per day, twice a day | Sitting in a chair for 5 min per day, twice a day | Sitting in a chair for 5 min per day, twice a day |
| Walking bedside (Walking training supported by a walker) | / | / | / | / | 2 min | 2 min | 2 min |
| **Goals** | | | | | | | |
| An increase in heart rate relative to resting heart rate | 5–15 beats per min | 5–15 beats per min | 20–30 beats per min | 20–30 beats per min | 20–30 beats per min | 20–30 beats per min | 20–30 beats per min |
| Borg score | / | <12 | 12-13 | 12-13 | 12–13 | 12–13 | 12–13 |
| PERME score | / | Sitting up in bed with a score of 3 | Meditation balance with a score of 3 | Sit-to-stand transfer or standing balance with a score of 3 | Bed-to-chair transfer with a score of 3 | Walking training with a score of 3 | / |

a Personalized cardiac rehabilitation training will be initiated based on the assessment after stabilization. Once patients meet the goals of the lower level, they can be assessed for the next level and begin the higher-level program.

b Muscle strength assessment was conducted using the Medical Research Council scale, ranging from level 0 to level 5, with higher levels indicating greater muscle strength.

NYHA classification = New York Heart Association classification of heart function; ROM = Range of motion; Borg score = Borg Rating of Perceived Exertion Scale; PERME score = Perme ICU mobility scores

© 2025 Wu L et al. *JAMA Network Open*.

**eTable 2.** Key Characteristics of Patients at CICU Administration

| | **Control (n=60)** | **Intervention (n=60)** | **Total (n=120)** |
|---|---|---|---|
| New York heart association functional class, No (%) | | | |
| III | 18 (30.0%) | 21 (35.0%) | 39 (32.5%) |
| IV | 42 (70.0%) | 39 (65.0%) | 81 (67.5%) |
| Blood pressure | | | |
| Systolic blood pressure, mean (SD), mm Hg | 140.9 (25.7) | 137.3 (27.5) | 139.1 (26.7) |
| Diastolic blood pressure, mean (SD), mm Hg | 84.3 (17.5) | 84.2 (15.3) | 84.2 (16.5) |
| Blood pressure >150/100 or <90/60 mmHg, No (%) | 23 (38.3%) | 27 (45.0%) | 50 (41.6%) |
| Fraction of inspired oxygen to achieve $SaO_2 \geq 92\%$, No (%) | | | |
| <40% | 35 (58.3%) | 28 (46.6%) | 63 (52.5%) |
| 40%-60% | 21 (35.0%) | 29 (48.3%) | 50 (41.6%) |
| >60% | 4 (6.6%) | 3 (5.0%) | 7 (5.8%) |
| N-terminal pro b-type natriuretic peptide, median (IQR), ng/L | 6195(2934-16052) | 5401(2906-9149) | 5733(2906-10326) |

CICU = Cardiac Intensive Care Unit.

© 2025 Wu L et al. *JAMA Network Open*.

**eTable 3.** Characteristics of Physical Therapy During CICU Stay[a]

| | Control (n=60) | Intervention (n=60) |
|---|---|---|
| Interval from CICU admission to randomization, median (IQR), hours | 39 (24-70) | 24 (24-48) |
| The number of physical therapy, median (IQR) [a] | 5 (5-5) | 5 (5-5) |
| Length of stay in CICU, median (IQR), days | 6 (5-8) | 6 (5-7) |
| Interval from hospital admission to discharge, median (IQR), days | 10.5 (8.2-14.7) | 11.0 (8.0-16.0) |
| The physical fitness levels at baseline, No (%)[b] | | |
| Level 1 | 50 (83.3%) | 29 (48.3%) |
| Level 2 | 10 (16.7%) | 19 (31.7%) |
| Level 3 | 0 | 11 (18.3%) |
| Level 4 | 0 | 1 (1.7%) |
| The physical fitness levels at final, No (%) [b] | | |
| Level 1 | 3 (5.0%) | 1 (1.7%) |
| Level 2 | 14 (23.3%) | 1 (1.7%) |
| Level 3 | 37 (61.7%) | 5 (8.3%) |
| Level 4 | 2 (3.3%) | 14 (23.3%) |
| Level 5 | 1 (1.7%) | 12 (20.0%) |
| Level 6 | 1 (1.7%) | 10 (16.7%) |
| Level 7 | 2 (3.3%) | 17 (28.3%) |

a Two patients in each group discontinued the assessment and intervention midway. The interval was calculated up to the point of discontinuation. For the control group, participants were assessed and received usual care.

b The physical fitness levels included seven levels, ranging from the weakest level 1 to the strongest level 7. The levels were assessed as indicated in the **eTable 1.**

CICU = Cardiac Intensive Care Unit.

© 2025 Wu L et al. *JAMA Network Open*.

**eTable 4.** Supporting Outcomes About Cardiopulmonary Function at CICU Discharge

| | Control (n=60) | Intervention (n=60) | Median difference (95%CI) [a] |
|---|---|---|---|
| Left ventricular Ejection fraction, median (IQR), % | | | |
| Baseline | 34 (27-45) | 36 (27-46) | |
| Final | 39 (30-53) | 37.3 (30-46) | -1.7 (-4.5 to 1.0) |
| Left ventricular ejection time, median (IQR), ms | | | |
| Baseline | 270.6 (214.8-324.8) | 258.2 (216.4-305.4) | |
| Final | 247.2 (200.1-329.8) | 285.3 (251.2-326.4) | 38.99 (3.33 to 68.47) |
| Cardiac output, median (IQR), L/min | | | |
| Baseline | 4.9 (4.0-6.1) | 5.0 (4.2-5.8) | |
| Final | 4.4 (3.8-5.1) | 4.69 (3.8-5.2) | -0.1 (-0.54 to 0.58) |
| Cardiac index, median (IQR), L/min/$m^2$ | | | |
| Baseline | 3.0 (2.4-3.7) | 2.9 (2.4-3.2) | |
| Final | 2.7 (2.3-3.2) | 2.6 (2.3-3.1) | -0.09 (-0.31 to 0.39) |
| Cardiac contractility index, median (IQR) | | | |
| Baseline | 114.6 (60.8-142.2) | 76.0 (52.0-107.0) | |
| Final | 92.2 (60.8-117.9) | 91.6 (53.5-131.9) | 17.49 (-4.91 to 59.74) |
| Early diastolic filling rate, median (IQR), % | | | |
| Baseline | 70.9 (54.7-89.6) | 71.7 (62.1-84.9) | |
| Final | 75.8 (61.8-92.4) | 70.2 (60.6-82.7) | -2.18 (-12.43 to 4.89) |
| Left heart work index, median (IQR), kg*m/$m^2$ | | | |
| Baseline | 3.7 (2.8-4.5) | 3.5 (2.8-4.3) | |
| Final | 2.8 (2.4-3.5) | 3.0 (2.3-3.5) | -0.08 (-0.42 to 0.52) |
| Systemic vascular resistance index, median (IQR), dyn.s/$cm^5$.$m^2$ | | | |
| Baseline | 2114.3 (1842.5-2795.7) | 2355.5 (2157.7-2606.7) | |
| Final | 2228.8 (1903.3-2616.2) | 2453.0 (2096.5-2940.8) | -31.4 (-156.91 to 118.93) |
| Ventricular systolic waves, median (IQR), | | | |
| Baseline | 107.2 (62.6-148.4) | 72.0 (48.7-103.7) | |
| Final | 94.4 (61.0-118.0) | 91.1 (49.9-126.2) | 15.1 (2.69 to 48.90) |
| Ventricular diastolic waves, median (IQR), | | | |

© 2025 Wu L et al. *JAMA Network Open*.

| | | | |
|---|---|---|---|
| Baseline | 23.0 (11.2-41.9) | 28.0 (16.7-46.2) | |
| Final | 20.7 (9.4-34.2) | 20.4 (8.1-40.6) | -12.2 (-23.9 to 1.5) |
| N-terminal pro b-type natriuretic peptide, median (IQR), ng/L | | | |
| Baseline | 5695 (2873-21536) | 3814 (2113-10136) | |
| Final | 2515 (1133-9204.15) | 1735 (897-3787) | 1414.5 (-200.07 to 3531.73) |
| High-sensitive cardiac troponin T, median (IQR), ng/L | | | |
| Baseline | 52.1 (30.7-175.0) | 54.5 (30.0-102.0) | |
| Final | 55.8 (24.23-120.9) | 46.9 (25.3-110.3) | 6.09 (-4.95 to 13.87) |
| High-sensitive C-Reactive Protein, median (IQR), mg/L | | | |
| Baseline | 18.2 (5.6-48.5) | 7.8 (2.7-22.6) | |
| Final | 10.7 (4.1-25.5) | 4.6 (1.9-20.5) | 2.96 (-0.16 to 8.63) |
| Forceful expiratory spirometry, median (IQR), L | | | |
| Baseline | 1.3 (1.0-1.7) | 1.7 (1.1-2.1) | |
| Final | 1.5 (1.1-2.0) | 1.8 (1.3-2.3) | -0.06 (-0.31 to 0.25) |
| Forced Expiratory Volume in one second to Forced Vital Capacity ratio, median (IQR) | | | |
| Baseline | 0.76 (0.67-0.88) | 0.76 (0.67-0.90) | |
| Final | 0.77 (0.67-0.87) | 0.83 (0.73-0.880 | 0.03 (-0.03 to 0.09) |
| Maximum minute ventilation, median (IQR), L/min | | | |
| Baseline | 24.5 (17.0-35.0) | 24.9 (16.9-41.5) | |
| Final | 24.0 (17.0-38.2) | 30.5 (20.6-47.4) | 5.3 (-0.6 to 9.9) |

a The median difference, along with its 95% CI, was calculated using the Wilcoxon rank-sum test to assess changes from baseline to final values.

CICU = Cardiac Intensive Care Unit; CI = Confidence interval.

© 2025 Wu L et al. *JAMA Network Open*.

**eTable 5.** Subgroup Analysis of the PERME Score

| Subgroup | Control | Intervention | Median difference (95%CI) [a] |
|---|---|---|---|
| **Age, No** | | | |
| ≤70 years | 27 | 36 | 6 (1 to 8) |
| >70 years | 33 | 24 | -0.5 (-2.99 to 2.99) |
| **Smoking history, No** | | | |
| Yes | 33 | 36 | 1.5 (-1.79 to 3.4) |
| No | 27 | 24 | 4.5 (1.2 to 8) |
| **Gender, No** | | | |
| Female | 22 | 18 | 4 (0 to 9) |
| Male | 38 | 42 | 2.2 (-0.79 to 3.999) |
| **$PaO_2/FiO_2$, No** | | | |
| ≥300 mmHg | 30 | 28 | 5 (0 to 6.99) |
| <300 mmHg | 30 | 32 | 2 (-0.79 to 4.59) |
| **Etiology, No** | | | |
| ICM | 34 | 27 | 2 (0 to 5) |
| non-ICM | 26 | 33 | 4.2 (-0.79 to 5.99) |

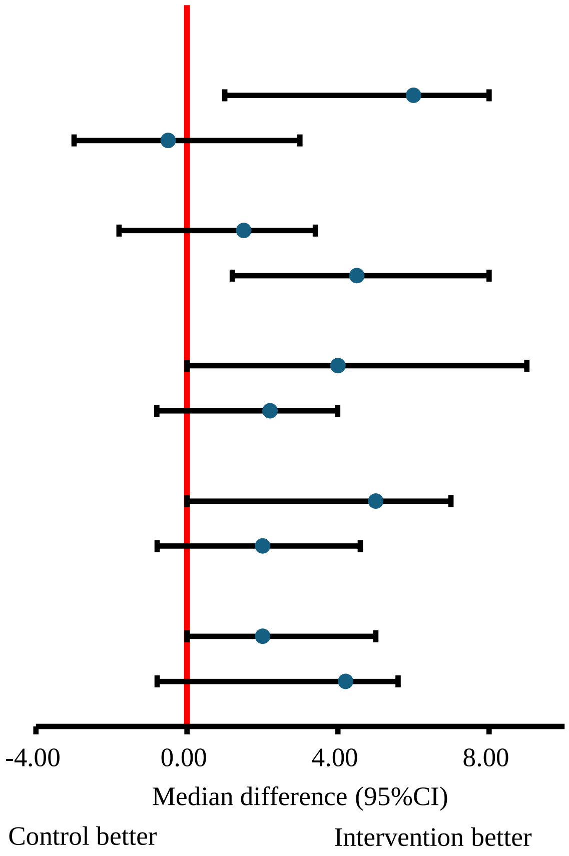


a The median difference and 95% CI were calculated using the Wilcoxon rank-sum test to assess changes from baseline to final values.

CI = confidence interval; ICM = ischemic cardiomyopathy; PERME = Perme ICU Mobility; $PaO_2/FiO_2$ = ratio of arterial partial pressure of oxygen to fraction of inspired oxygen.

© 2025 Wu L et al. *JAMA Network Open*.
