# Supplementary material for: Early Cardiac Rehabilitation for Critically Ill Patients With Acute Decompensated Heart Failure: A Randomized Clinical Trial
Source: JAMA Netw Open. 2025 Jul 30;8(7):e2524141. doi: 10.1001/jamanetworkopen.2025.24141 (PMC12311714; doi:10.1001/jamanetworkopen.2025.24141)
Supplement: Supplement 3. — Data Sharing Statement [file jamanetwopen-e2524141-s003.pdf]

Data Sharing Statement

Wu. Early Cardiac Rehabilitation for Critically Ill Patients With Acute Decompensated Heart Failure. *JAMA Netw Open*. Published July 30, 2025.
doi:10.1001/jamanetworkopen.2025.24141

Data
Additional Information: Chinese Clinical Trial Registry (ChiCTR2100050151).
Data available: Yes
Data types: Deidentified participant data
How to access data: Data from this paper are available upon request from the lead contacts: Cuilian Dai (email: daicl@xmu.edu.cn) and Binbin Liu (email: liubinbin@xmu.edu.cn).
When available: With publication

Supporting Documents
Document types: Statistical/analytic code
How to access documents: Data from this paper are available upon request from the lead contacts: Cuilian Dai (email: daicl@xmu.edu.cn) and Binbin Liu (email: liubinbin@xmu.edu.cn).
When available: With publication

Additional Information
Who can access the data: researchers whose proposed use of the data has been approved
Types of analyses: for a specified purpose
Mechanisms of data availability: with investigator support
